# Processing, structure, and properties of PLA/PHA blended melt-blown nonwoven webs for filter media applications

**DOI:** 10.1007/s10853-026-13145-5

**Published:** 2026-06-24

**Authors:** Avik Kumar Dhar, Ivan Moldavchuk, Maitry Bhattacharjee, Joe Nageotte, Gajanan Bhat, Sudhagar Mani

**Affiliations:** 1 Department of Textiles, Merchandising, and Interiors, University of Georgia, 321 Dawson Hall, 305 Sanford Drive, Athens, GA 30602 USA; 2 School of Chemical, Materials, and Biomedical Engineering, University of Georgia, 155B, 110 Riverbend Road, Athens, GA 30605 USA

## Abstract

Melt-blown nonwovens used in respiratory protection are predominately made from non-biodegradable polypropylene (PP), raising sustainability concerns. This study developed bio-based, biodegradable melt-blown nonwovens using polylactic acid (PLA) and polyhydroxyalkanoate (PHA) blends for efficient PM_0.3_ filtration. The effects of key processing parameters (air pressure, melt temperature, and blend ratio) on fiber morphology, thermal behavior, filtration, and mechanical properties were evaluated. The fiber diameters of developed webs ranged from 3 to 7 µm, and the incorporation of PHA significantly increased fiber diameters. The glass transition temperature decreased from 62.9 °C (pure PLA) to 58.3 °C (90/10-PLA/PHA), while melting temperature showed a slight reduction, indicating minor changes in crystallinity. The developed webs achieved > 96% PM_0.3_ removal efficiency and < 51 Pa pressure drop after corona charging (− 50 kV). Furthermore, PLA/PHA blended webs also showed improved mechanical performance, with ~ 14.1% higher maximum force and ~ 27.6% greater strain at maximum load. These results demonstrate that PLA/PHA melt-blown nonwovens are promising biodegradable alternatives to PP-based filter media.

**Graphical abstract:**

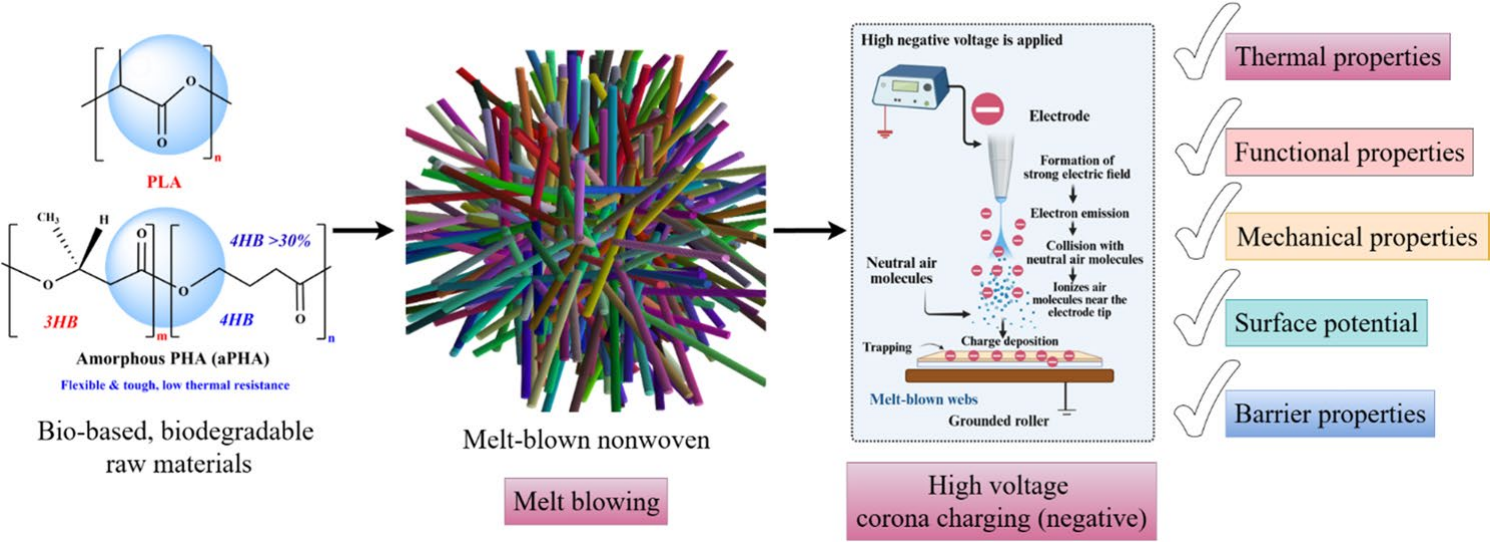

**Supplementary Information:**

The online version contains supplementary material available at 10.1007/s10853-026-13145-5.

## Introduction

Particulate matter (PM), a primary form of anthropogenic air pollutant, is one of the most harmful airborne pollutants due to its tiny size and high adhesion to other toxic substances. Rapid industrialization and urbanization have exacerbated air pollution, introducing more PM into the atmosphere [1]. The presence of PM in the air poses significant risks to human health due to inhalation of toxic substances. Long-term exposure to higher PM levels escalates the risk to lung health [2]. Furthermore, the prevalence of a variety of pathogens (bacteria, viruses, and microscopic organisms) in the air stream has posed a major threat to both the environment and human health. Recently, we have experienced the COVID-19 pandemic, which caused huge respiratory illnesses and fatalities globally due to the rapid transmission of the coronavirus (SARS-CoV-2) by airborne virus-laden droplets [3]. To address the health hazards posed by these harmful particles, high-efficiency air filters have been widely utilized to safeguard humans [4, 5].

Melt-blown nonwovens have received tremendous research and industrial attention for air filtration due to their three-dimensional structure, random fiber arrangement, large specific surface area, high porosity, small pore sizes, lightweight, commendable air permeability, low-pressure drop, and high barrier performance [6, 7]. Additionally, applying electret treatments (triboelectrification, hydrocharging, corona charging, etc.) allows melt-blown nonwovens to easily acquire electrical charges, which enhance their ability to efficiently capture fine particles through electrostatic interaction. Hydrocharging can achieve the highest filtration efficiency, but it is a complex phenomenon, requires separate equipment, and uses high-pressure pure water, making it costly. In contrast, corona charging is simpler, less expensive, and can be integrated directly into the melt-blown production line [8–10]. Furthermore, the filtration efficiency of the negatively charged filter is higher than the positively charged web due to a substantially higher concentration of negative ions produced during corona charging [11]. In addition, negative air ions have greater mobility in an electric field than positive ions [12, 13].

Traditional fibrous melt-blown nonwoven webs comprise polymers from petrochemical raw material sources (PP, PE, and PET) because of their easy processability, low cost, high melt flow rate, low melt viscosity, and chemical stability. Yet, they persist in the environment for a long time and are challenging to break down, which unavoidably contributes to the sharp rise in plastic and microplastic pollution [14–16]. These plastic materials usually take at least 200 years to fully degrade, and their incomplete breakdown produces micro- and nanoplastics that contaminate air, soil, and oceans, causing significant global pollution [17].

To lessen dependence on petrochemical resources and encourage eco-friendly, sustainable energy development, there has been a growing interest in using bio-based polymers, especially PLA and PHA, which have gained significant attention. Some studies have been conducted so far on developing bio-based melt-blown nonwovens [18–22]. For example, Xu et al. [23] fabricated hydrocharged melt-blown nonwovens (PLA/EBS/BaTiO_3_) with excellent performance (filtration efficiency—99.69% for PM_0.3_, pressure drop—27.44 Pa) [23]. Zhang et al. [18] implemented a commercially viable liquid electret PLA melt-blown nonwovens with > 95% filtration efficiency and excellent charge stability (> 1 year) [18]. However, there is limited research that investigated the structure–properties relationship and barrier properties of bio-based blended nonwoven webs. For instance, recently, Liu et al. [24] developed biodegradable PLA/PHBV melt-blown nonwovens with high strength and excellent toughness [24]. Meng et al. [25] fabricated PLA/PBS melt-blown nonwovens with excellent mechanical properties (strength 2.19 MPa and strain 12.97%) [25]. To the best of the author’s knowledge, there has been no comprehensive study on the fabrication of PLA/PHA blended melt-blown nonwovens and the investigation of their structure–property relationships for air filtration applications.

Therefore, current research is designed to provide an in-depth understanding of the melt-blown processing of bio-based, biodegradable PLA/PHA blended nonwoven webs for their potential application in air filtration. Physical properties, surface morphology, fiber diameter, mechanical, thermal, and barrier properties were analyzed at different processing conditions to investigate the optimum processing conditions. Furthermore, developed webs were treated with high-voltage negative corona to impart electrostatic charges. Findings of this study will provide insight into processing, structure–properties relationships, and barrier performance of bio-based biodegradable blended melt-blown nonwovens for air filtration.

## Experimental section

### Materials

**Figure 1:**
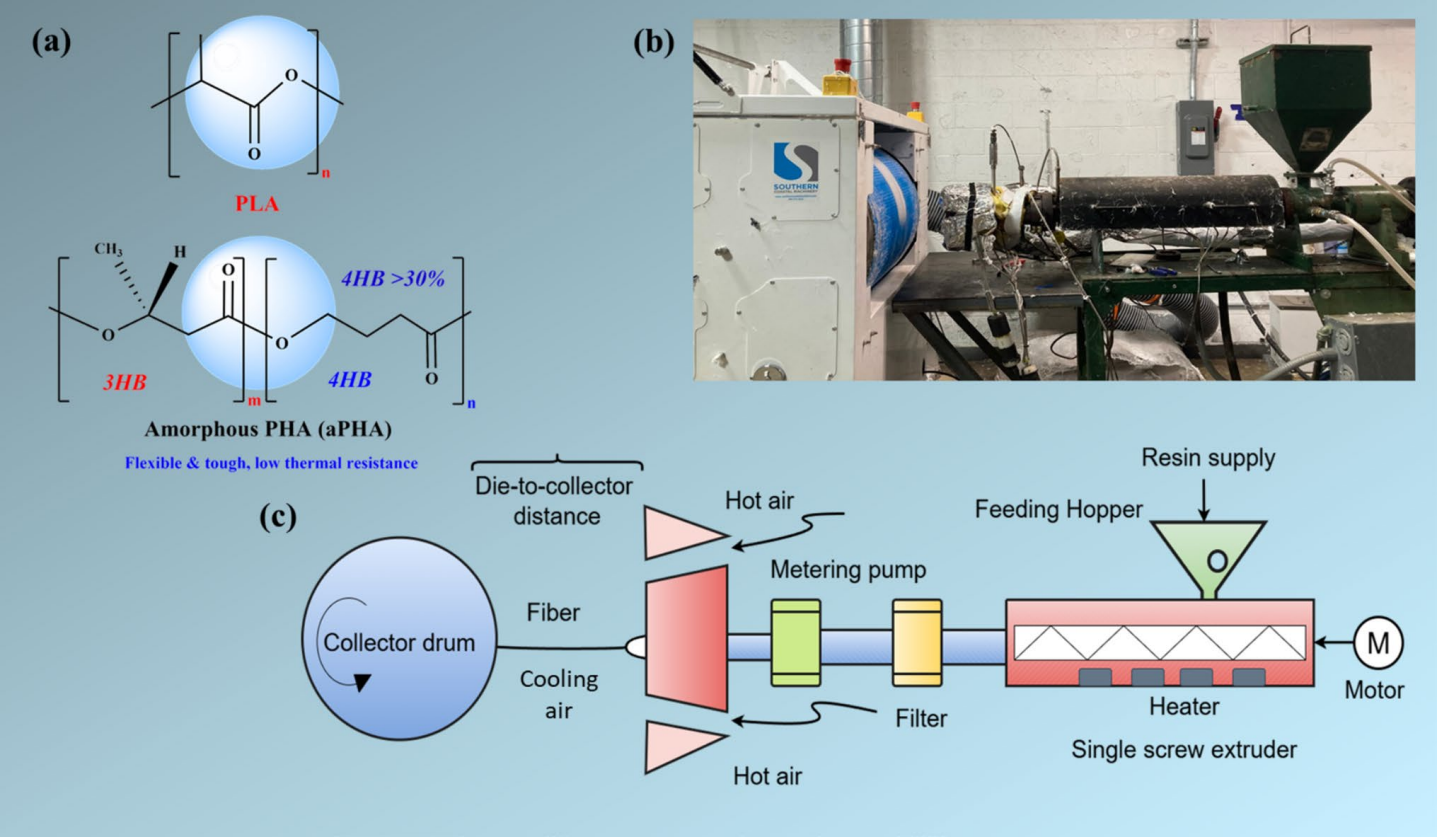
**a** Chemical structures of PLA and amorphous PHA used in this research **b** melt-blown production line (15 cm wide) at the university of Georgia **c** schematic diagram of melt blowing.

The 100% PLA resin used in this study was Ingeo™ Biopolymer 6252D, a melt-blown grade resin derived from renewable resources developed by NatureWorks LLC (USA). For blending PHACT TM MA1250P master batch supplied by CJ Biomaterials was used. MP1250P is a masterbatch synthesized from a blend of PLA and amorphous PHA (aPHA). The aPHA component was the PHACT A1000P grade from CJ Biomaterials (USA), while the PLA was Ingeo 4032D from NatureWorks LLC. The mixture contains 55% PLA and 45% aPHA by weight. Table 1 summarizes the basic specifications of the raw polymers used in this research. Figure 1a represents the structure of PLA and PHA used in this research.

**Table 1:** Specification of PLA and PHA used in this research

Properties	PLA (100% PLA)	PHA—masterbatch (55/45-PLA/PHA)
Commercial grade	Ingeo 6252D	PHACT MA1250P
Supplier	NatureWorks LLC (USA)	CJ Biomaterials (USA)
Melt index, g/10 min, (2.16 kg)	70–85 (210 °C)	5 ~ 8 (190 °C)
Glass transition temperature (°C)	55–60	− 17, 60
Crystalline melt temperature (°C)	155–170	150 ~ 170
Specific gravity (D792)	1.24	1.22

### Melt-blown processing

Before processing, PLA (6252D) resin was dried in a convection oven (DNYSYSJ, China) at 80 °C for 5 h, and PHACT TM MA1250P masterbatch was dried at 75 °C for 4 h to remove moisture to avoid hydrolysis during the melt-blown process. For blending PLA resin with masterbatch, a portable mixer from BILT HARD (capacity-3-½ cubic ft., motor: 1750 RPM, 120 VAC/60 Hz/3.5 A, 38 cm drum opening) was used. The dried PLA and PLA/PHA masterbatch were used to develop PLA and PLA/PHA blended (95/5 and 90/10-PLA/PHA) nonwovens with varying basic weight under the conditions of melt/die temperature − 235 °C, air temperature − 260 °C, DCD − 14 cm, and collector drum speed (rpm) − 3. Melt blowing was conducted on a 15 cm (6 inches) – wide pilot line at the University of Georgia, Athens, Nonwovens Material Research Lab using Exxon dies with ten holes per centimeter in the process (0.28 g/h/m throughput). However, before introducing PLA, the system was purged appropriately to prevent contamination and associated spinning problems. Three different air pressures (55, 72, and 88 kPa) were used to develop nine different webs. Table 2 summarizes the sample types with respective codes.

**Table 2:** Processing conditions, sample specifications, and physical properties of PLA/PHA blended melt-blown nonwoven webs

Sample type	Sample code	Air pressure (kPa)
100% PLA	100–55	55
	100–72	72
	100–88	88
95/5-PLA/PHA	95/5–55	55
	95/5–72	72
	95/5–88	88
90/10-PLA/PHA	90/10–55	55
	90/10–72	72
	90/10–88	88

In brief, PLA and PLA/PHA blended melt-blown webs were produced as follows: drying of polymer chips, mixing, and feeding into the screw extruder, followed by heating and melting. Next, the melt passed through a filter and a metering pump in turn and was finally forced through the spinneret holes. The molten filaments were drawn by hot air, cooled, and nonwoven samples were collected on a rotating drum. Figure 1b and c depicts the melt-blown production line and schematic diagram.

### Corona charging

The developed webs were corona charged to impart electrostatic properties. The filtration efficiency of the negatively charged filter is higher than the positively charged web [11]. This is because the concentration of negative ions generated by corona charging is much higher than that of positive ions. Furthermore, negative air ions have greater mobility in an electric field than positive ions [12, 13]. Therefore, the developed webs were charged using a dual-bar SIMCO Chargemaster negative corona charging equipment. The corona charging speed was 2 m/min, with the distance between corona and fabric set to 7 cm, and the applied voltage was –50 kV. The corona electret melt-blown nonwovens were rolled onto tubes. Additionally, this process was carried out at room temperature (25 ± 2 °C) with a relative humidity of about 45 ± 5%. During the charging process, nonconductive nitrile gloves were used for handling the fabrics to avoid any adverse effect on their properties. After charging, the charged fabric was stored at room temperature in a black polybag to avoid direct contact with daylight to allow long-term storage without any adverse effect on its properties until further analysis.

### Characterization

#### Melt flow rate (MFR) of polymers

The MFR of the polymers is a critical property in the melt-blown process, and it directly influences the processability and web quality. MFR of the polymers were determined by using the Tinius Olsen MP1200 melt flow tester using orifice diameters of 0.1049 cm and 0.3988 cm length, with a preheat time of 60 s (at 210 °C and 2.16 kg load).

#### Basis weight and thickness

The basis weights of the developed webs, measured in grams per square meter (g/m^2^), were determined according to the ISO 3801 standard. Ten samples, each 100 cm^2^, were randomly selected, weighted, and averaged. Fabric thickness was measured in 20 random locations using a ProGage thickness tester (Thwing-Albert Instrument Company, USA) according to ASTM D1777-96, with the average thickness values reported.

#### Fourier transform infrared spectroscopy (FTIR)

The change in functional group after blending PHA with PLA was investigated through FTIR analysis. FTIR analysis was performed using Perkin Elmer Spectrum TM 3 (USA) at an ambient temperature of 25 °C. The samples were analyzed within a wavelength range of 4000–650 cm^−1^ at a resolution of 4 cm^−1^ and averaged over 32 scans.

#### Differential scanning calorimetry (DSC)

The DSC 8000 differential scanning calorimeter from PerkinElmer (exclusive double-furnace DSC) was used to investigate the thermal properties of the developed materials at different blend ratios. About 5–6 mg of samples were prepared in the standard crucible. Thermal scanning was performed through heating–cooling cycles. After each cycle, the sample was held for 2 min. Heating was conducted from 25 to 230 °C in an N_2_ atmosphere (flow of 100 mL/min) at a rate of 10 °C/min. After holding for 2 min, the sample was cooled to 25 °C at a rate of 10 °C/min. Then, the temperature was again raised to 230 °C at a rate of 10 °C/min, to obtain the second heating curve and subsequently cooled to room temperature. The second heating run was used to evaluate the thermal properties of the tested materials after removing the thermal histories by melting and cooling under controlled conditions. The crystallization enthalpy (∆*H*_c_), glass transition temperature (*T*_g_), melting temperature (*T*_m_), and melting enthalpy (∆*H*_m_) of the samples could be obtained from second heating curves. The relative degree of crystallinity (*X*_c_) was calculated using the following equation:

$${X}_{\mathrm{c}}=\frac{{\Delta H}_{\mathrm{m}}-{\Delta H}_{\mathrm{c}}}{{\Delta H}_{0}.C}$$

Here,

${\Delta H}_{0}$ is the fusion enthalpy of 100% crystalline PLA (93 Jg^−1^) and C is the fraction of PLA in the blend [26, 27].

#### X-ray diffraction measurements (XRD)

The crystal structure of the pure PLA and PLA/PHA blended webs was characterized using X-ray diffractometry (Bruker D2 PHASER), radiation source, Cu- Kα (λ = 1.54184); scan range − 10 to 80 degrees 2 theta, step increment − 0.01 degrees 2 theta, scan speed − 0.15 s/step. A single layer of webs was cut and placed on the Si wafer (zero background). Sample mass was kept at 0.0552–0.0761 g.

#### Thermal gravimetric analysis (TGA)

The thermal stability of the developed webs was determined using a thermogravimetric analyzer (TGA 8000, PerkinElmer, USA). The effect of blend ratio on the thermal stability of webs was investigated through this analysis. Samples of 5–6 mg mass were used in a ceramic sample pan and were scanned from 25 to 600 °C at a heating rate of 10 °C/min under N_2_ atmosphere. For each sample, three replicates were done, and the average onset temperature was reported.

#### SEM images and fiber diameters

The surface morphology of the developed webs was investigated using a scanning electron microscope (Field Emission SEM, Thermo Fisher Scientific –FEI, Teneo, USA) under 5–10 kV voltages and a various magnification of 500 × , 1000 × , or 1500 × . The samples were coated with gold (SPI-Module Sputter coater, USA) for 120 s before SEM analysis. Fiber diameters and their size distribution were determined using ImageJ software, with the average diameter calculated from a minimum of 100 individual measurements at different positions from multiple SEM images.

#### Air permeability

Air permeability of the developed webs was determined using a TEXTEST FX3330 air permeability tester (Switzerland), according to ASTM D73796, with an area specimen of 38 cm^2^ and a pressure of 120 Pa. Ten replicates were tested for each condition, and an average was recorded for each sample with a standard deviation.

#### Pore size distribution

The pore structure (mean flow pore diameter in microns) and pore size distribution in developed webs were analyzed by a CFP-1100AEX capillary flow porometer (Porous Materials Inc., USA) according to the ASTM F316-03 (2011) method (dry up—wet up). Samples were cut into a circle with a diameter of 1.5 cm and then placed in the instruments. GalWick (surface tension − 15.9 dynes/cm) was used as the wetting liquid.

#### Surface potential

The output performance (surface potential/static charge) of the as-produced and corona-charged webs was measured using a non-contact Electrostatic field meter (FMX-004, SIMCO-Ion, USA) and reported in kV. The tests were performed at a room temperature of 25 ± 2 °C and a relative humidity of 45 ± 5%. Ten measurements were taken at various locations on the web for each sample, and the average is reported with standard deviations.

#### Filtration efficiency & pressure drop

An automated filtration efficiency tester (TSI 8130A, TSI Instruments Co., Ltd., USA) was used to evaluate the filtration performance of melt-blown nonwovens. NaCl (2% salt solution in distilled water; 99% purity) was used to generate aerosol particles (aerosol diameter between 0.2 to 0.4 μm). In this process, aerosols generated by the aerosol generator move to the top filter holder and are drawn through the filter by a vacuum applied downstream. Two laser diode light-scattering photometers then measure aerosol concentrations before and after the filter, with a sensitivity of 0.0001%.

The filtration efficiency and pressure drop were calculated as follows:

$$E = \left(1-\frac{{C}_{\mathrm{d}}}{{C}_{\mathrm{u}}}\right)\times 100$$

where E is the filtration efficiency of the fiber filter membrane, %.

${C}_{\mathrm{d}}$ and ${C}_{\mathrm{u}}$ represent the number of particles in the filtered upstream and downstream, respectively. The test was conducted under ambient conditions (room temperature of 25 ± 2 °C and relative humidity of 45 ± 5%).

Highly accurate electronic pressure transducers and a TSI® Model 4045 Mass Flow Meter determined filter resistance and flow, respectively. Pressure transducer (differential pressure across the filter) readings were taken between every test to determine the zero offsets and background values.

$$\Delta P = {P}_{1}-{P}_{2}$$

where Δ*P* is the pressure drop, Pa;

${P}_{1}$ is the pressure before filtration;

${P}_{2}$ is the pressure after filtration, Pa.

The differential pressure transducer had a nominal linear output with a standard range of 0–2.5 kPa. For each sample, measurements were made in ten different places at an airflow rate of 32 L/min, and an average of 10 measurements were recorded.

Quality factor (QF) is a comprehensive evaluation of the filtration performance of filter materials. The larger the value of QF, the better the comprehensive performance. QF was calculated according to the equation:

$$\mathrm{Q}\mathrm{F} = \frac{-\mathrm{ln}\left(1-E\right)}{\Delta P}$$

where QF refers to the quality factor, Pa^−1^;

*E* is the filtration efficiency, %;

Δ*P* was reported in Pa, representing the filtration resistance.

#### Mechanical properties

The mechanical performance of the developed webs (pure and blended webs) was investigated by tensile test according to ASTM –5035–11(2019) using an Instron 4400 R (origin) tensile tester to determine the tensile modulus, strength, and elongation of the melt-blown nonwovens in the machine direction. Five samples of 150 mm × 25 mm in the machine direction were used with an initial gauge length of 75 mm and were tested at an extension rate of 100 mm/min until sample breakage. To ascertain accuracy, at least five replicates of each sample were used to get the average values that correspond to the standard deviations.

#### Statistical analysis

Statistical analysis was performed to evaluate the effect of air pressure on the performance characteristics of the three different types of samples (100% PLA, 95/5 blend, and 90/10 blend). For each condition, at least 10 replicates were performed to ensure statistical reliability. Descriptive statistical analysis ensured the calculation of the mean ($\overline{X }$), standard deviation (s), coefficient of variance, and confidence interval for each sample group at each air pressure level. Two-way ANOVA was performed to investigate the effect of air pressure and sample type on the dependent variables. Furthermore, the interaction effect between the independent variables was also investigated. Post hoc test was performed (Tukey’s HSD) to investigate which specific groups differ significantly at the level of significance of 0.05. The statistical analysis (ANOVA) of the data gathered from all designs was carried out using the JMP and RStudio tools.

## Results and discussions

### MFR of used polymers

MFR of the used polymers and their combinations are presented in Table 3. The average of three replicates was reported with standard deviation. ANOVA results demonstrated a significant difference among the four polymer compositions (*p* value = 1.14 × 10^–12^ ***). Post hoc Tukey test showed that 100% PLA had the highest MFR, 95/5-PLA/PHA and 90/10-PLA/PHA had significantly lower but statistically similar MFR, and 55/45-PLA/PHA had the lowest MFR, significantly lower than all other compositions. Decreasing MFR after adding aPHA to pure PLA is consistent with literature and can be explained by the increased complex melt viscosity from intermolecular interactions, especially at low to moderate shear rates [28, 29].

**Table 3:** MFR of the polymers used in this research (at 210 °C and 2.16 kg)

Polymer compositions	MFR (g/10 min)
100% PLA	73.19 ± 7.77
95/5-PLA/PHA	50.43 ± 3.18
90/10-PLA/PHA	47.73 ± 2.57
55/45-PLA/PHA	14.91 ± 1.98

### Basis weight and thickness

**Figure 2:**
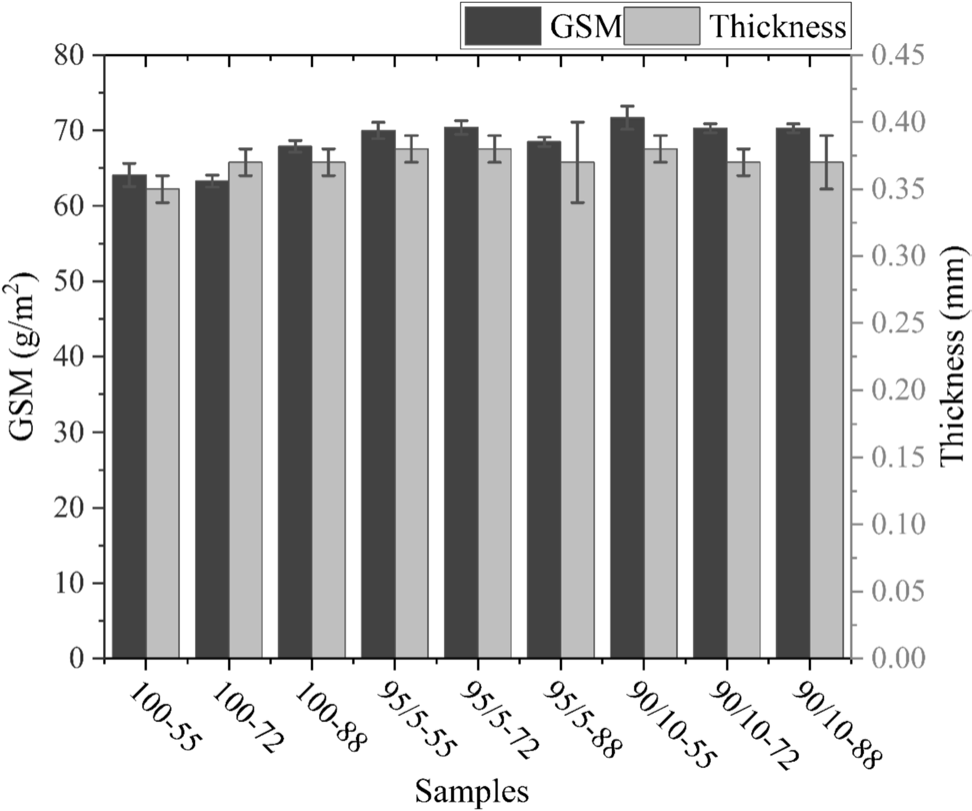
Basis weight and thickness of the developed webs.

The physical properties (basis weight and thickness) of the developed webs are depicted in Fig. 2**.** The goal was to maintain the basis weight in the 65–70 g/m^2^ range. Therefore, polymer throughput and collector drum speed were kept constant throughout the melt blowing process. All the samples had the expected basis weight, and the thickness values of all samples fell within the same range of 0.35–0.38 mm. The low standard deviation of the web’s weight and thickness indicates the formation of a regular web.

### Fourier transform infrared spectroscopy

**Figure 3:**
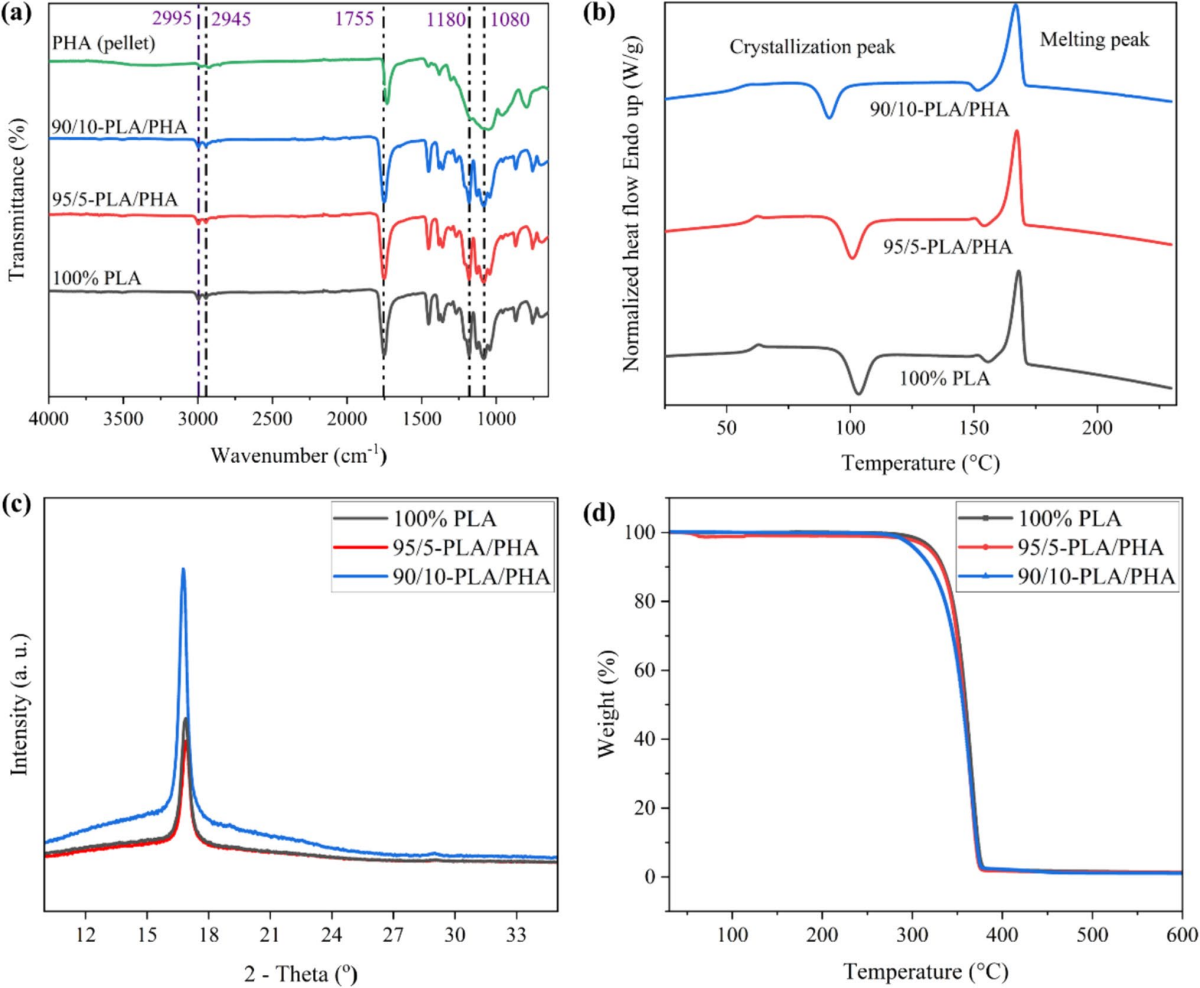
**a** FTIR spectrum **b** DSC curve (2nd heating) **c** Representative XRD profiles **d** Thermogravimetric analysis of PLA and PLA/PHA blended webs.

FTIR analysis provides a rapid, non-destructive, and reliable method to identify the chemical composition and structure of the fibers within the blend. All spectra (Fig. 3a) exhibited characteristics of the PLA functional group of bands. Due to the low PHA content in the PLA/PHA blends, PHA-specific bands were indistinguishable, resulting in no significant spectral difference between PLA and PLA/PHA blended webs. In FTIR spectra of 100% PLA, key peaks appeared at approximately 1750–1760 cm^−1^ and 1180 cm^−1^, corresponding to the C=O stretching and C–O–C stretching vibration, respectively. Furthermore, the peaks at 2995 and 2945 cm^−1^ were associated with the asymmetrical and symmetrical stretching vibration of the –CH_3_ groups, respectively [30]. Other peaks observed are: –OH bend at 1044 cm^−1^, –C–O stretch at 1129 cm^−1^ and 1083 cm^−1^, and C–C stretch of the PLA crystalline phase at 869 cm^−1^ [31].

On the other hand, 100% PHA also demonstrates a strong ester C=O peak, but it appears slightly lower in wavenumber (within the range of 1715–1740 cm^−1^). This finding was supported by the literature [32, 33]. Therefore, incorporation of PHA shifted the C=O peak to the lower wavenumber. However, the lack of band shifts suggested that no detectable chemical reaction occurred between PLA and PHA during the process; the polymers were physically blended. Lopresti et al. [34] also developed polymeric scaffolds from PLLA/PHA via the thermally induced phase separation method and observed no band shift after blending [34].

### Differential scanning calorimetry

DSC analysis of 100% PLA and PLA/PHA blended nonwoven provides insight into their thermal properties, including glass transition temperature (*T*_g_), crystallization, and melting behavior. Figure 3b demonstrates the thermogram of PLA and PLA/PHA blended melt-blown nonwovens for the 2nd heating cycle.

The *T*_g_ decreased with the incorporation of PHA content (from 62.9 °C for pure PLA to 58.3 °C for 90/10-PLA/PHA). PHA acted as a plasticizer to increase the chain mobility of PLA and acted as a nucleating agent as the blend crystallized at lower temperatures. Furthermore, *T*_c_ dropped significantly with increasing PHA content (from 103.3 to 91.5 °C). The crystallization temperature (*T*_c_) was indicative of the polymer’s crystal growth rate and crystallization ability; a lower *T*_c_ signified enhanced crystal growth and improved crystallization capability [35].

Mondragón-Herrera et al. [28] observed 3 °C and 5 °C reduction of *T*_g_ and *T*_c_ due to incorporation of PHA ~ 12% by wt. in PLA. Enthalpy of crystallization (∆*H*_c_) decreased with increasing PHA content (28.1 for 95/5-PLA/PHA and 21.4 for 90/10-PLA/PHA), which suggests a lower degree of crystallization. This reduction was due to the presence of a smaller amount of PLA in the composition and the amorphous nature of PHA. However, the melting temperature decreased slightly with more PHA (from 168.2 to 166.9 °C), indicating PHA slightly disrupted the crystal structure of PLA, lowering the melting point [28].

Additionally, the observed shoulder before the sharp melting peak corresponds to the transition or recrystallization of the disordered α΄-crystal with loose and disordered chain packing to the more ordered α-crystal form during heating. In this study, a similar shoulder peak was observed immediately preceding the PLA melting peak, as shown in Fig. 3b. With the incorporation of PHA, this small shoulder slowly disappeared, suggesting the possible nucleation effect of PLA [36]. Olejnik et al. [37] also explored that only a small addition of PHB was useful for reducing the melting point, which corresponded to the material processing temperature and acted as a plasticizer for PLA processing [37]. Furthermore, the degree of crystallinity of the blended webs increased from 8.1% for pure PLA to 20.6% for 90/10-PLA/PHA due to the increasing content of PHA, even though PHA was amorphous. The increased crystallinity indicated that the presence of aPHA enhanced the mobility of the polymer chains, which facilitated the alignment and packing of PLA chains into crystalline domains [38].

### X-ray diffraction measurement (XRD)

Figure 3c illustrates the XRD pattern of pure PLA and PLA/PHA blended webs. 100% PLA showed a broader but distinct peak at around 16.7° (2*θ*), which is the characteristic peak of the (200/110) planes of the α-crystalline form of PLA. The overall intensity was moderate, which reflected the semi-crystalline nature of PLA. The presence of this peak for all three samples indicated that PLA was the dominant. In contrast, for 95/5-PLA/PHA, the main peak position remained like pure PLA but was slightly more intense and sharper than that of pure PLA. This further confirmed the increase in crystallinity (as observed in DSC), due to PHA acting as a nucleating agent, promoting the nucleation and crystallization rate, leading to more ordered PLA crystal formation. In addition, PHA also contributed to the enhanced mechanical properties of blended webs. However, for 90/10-PLA/PHA, the peak was the most intense and sharpest among the three samples, which indicated further increase in crystallinity with increasing PHA content. The higher intensity and narrower width of the peak reflected larger or more perfect crystalline domains in the blend [35].

### Thermogravimetric analysis

Figure 3d demonstrates the thermogravimetric analysis curve of 100% PLA and PLA/PHA blended melt-blown nonwoven webs. 100% PLA had slightly higher thermal stability than the PLA/PHA blended samples. The incorporation of PHA (inherently lower thermal degradation temperature) slightly lowered the thermal stability of blends, especially at higher concentrations (10% PHA). Amorphous PHA has lower thermal stability compared to pure PLA. Therefore, the amorphous PHA components began to degrade at a lower temperature, initiating mass loss in the blend before the PLA component degrades. This reduced thermal stability of the polymer matrix and accelerated thermal degradation compared to pure PLA [28, 39].

### SEM images and fiber diameters

**Figure 4:**
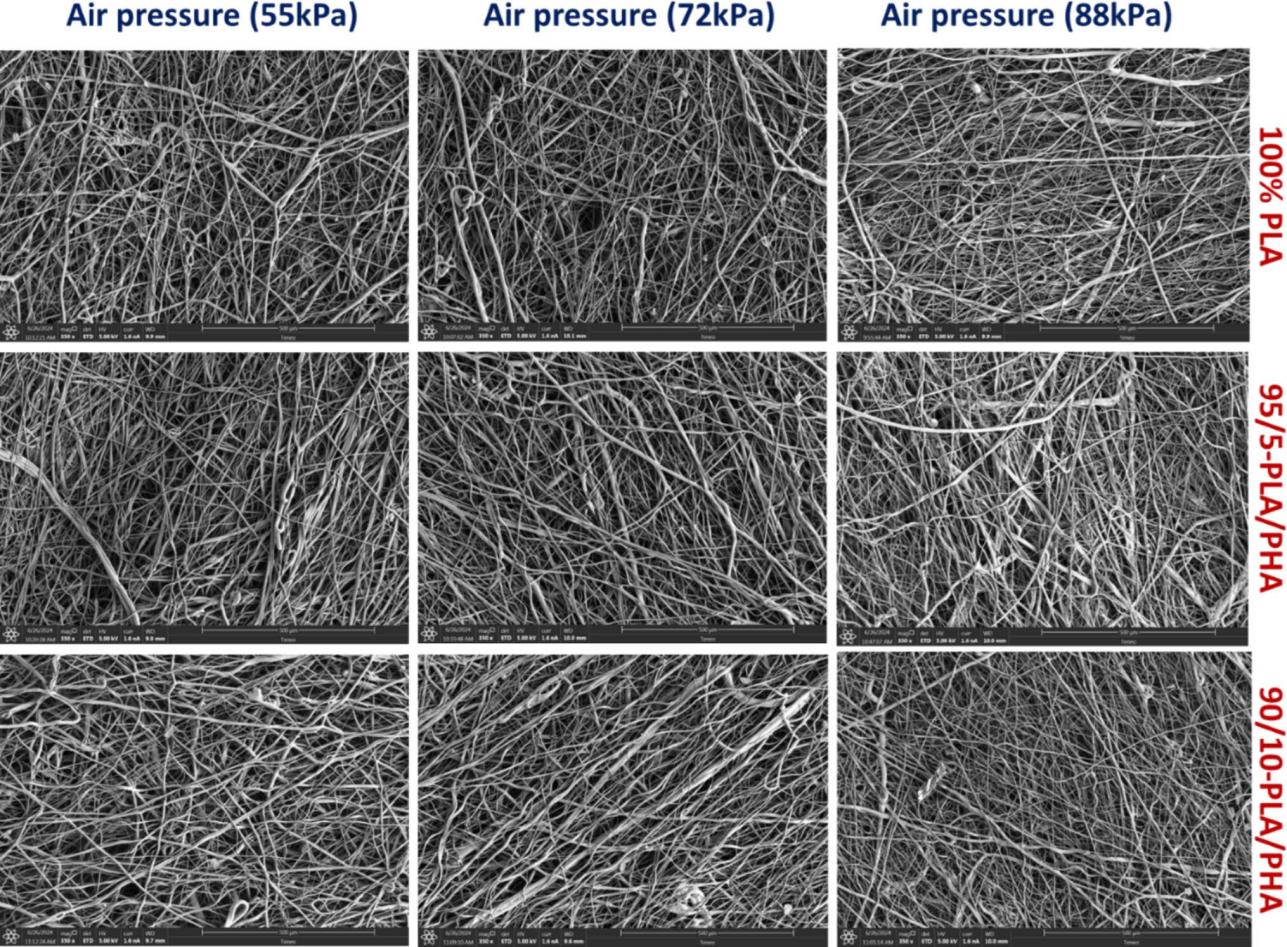
Surface morphology of PLA/PHA blended webs** (**SEM images, magnification—350 × , ETD detector).

SEM was used to observe the web structure and to determine the fiber diameters. The representative SEM photographs of webs of different compositions are shown in Fig. 4. Melt-blown webs were randomly arranged, and fiber diameter ranged from 3 to 7 μm. Increasing air pressure consistently resulted in smaller and more uniform fiber diameters across all compositions. Higher air pressure increased the drag force on molten polymer strands, stretching them into thinner fibers.

Figure S1 is the histogram illustrating the distribution of fiber diameters for different polymer compositions and air pressures. At 55 kPa air pressure, the fiber diameter of 100% PLA was widely spread, with peaks around 2.5–3.5 μm, but with some larger diameters. However, at 72 kPa, the distribution shifted slightly to the left, showing a more concentrated peak around 3–4 μm. At 88 kPa, the fiber diameters became smaller and more uniform, with most values around 2.5–3.5 μm. This finding supported the idea that higher air pressure leads to finer and more uniform fibers.

Furthermore, the incorporation of PHA resulted in an overall broader fiber diameter distribution, although higher air pressure produced a relatively narrower distribution. At 55 kPa, the fiber distribution of 95/5-PLA/PHA demonstrated a broad distribution with multiple peaks, ranging up to 12 μm. At 72 kPa, the distribution remained wide but slightly more concentrated. However, at 88 kPa, the fiber diameters became more uniform and significantly smaller, mostly within the 2–5 μm range. A similar trend was observed for 90/10-PLA-PHA webs as well.

**Figure 5:**
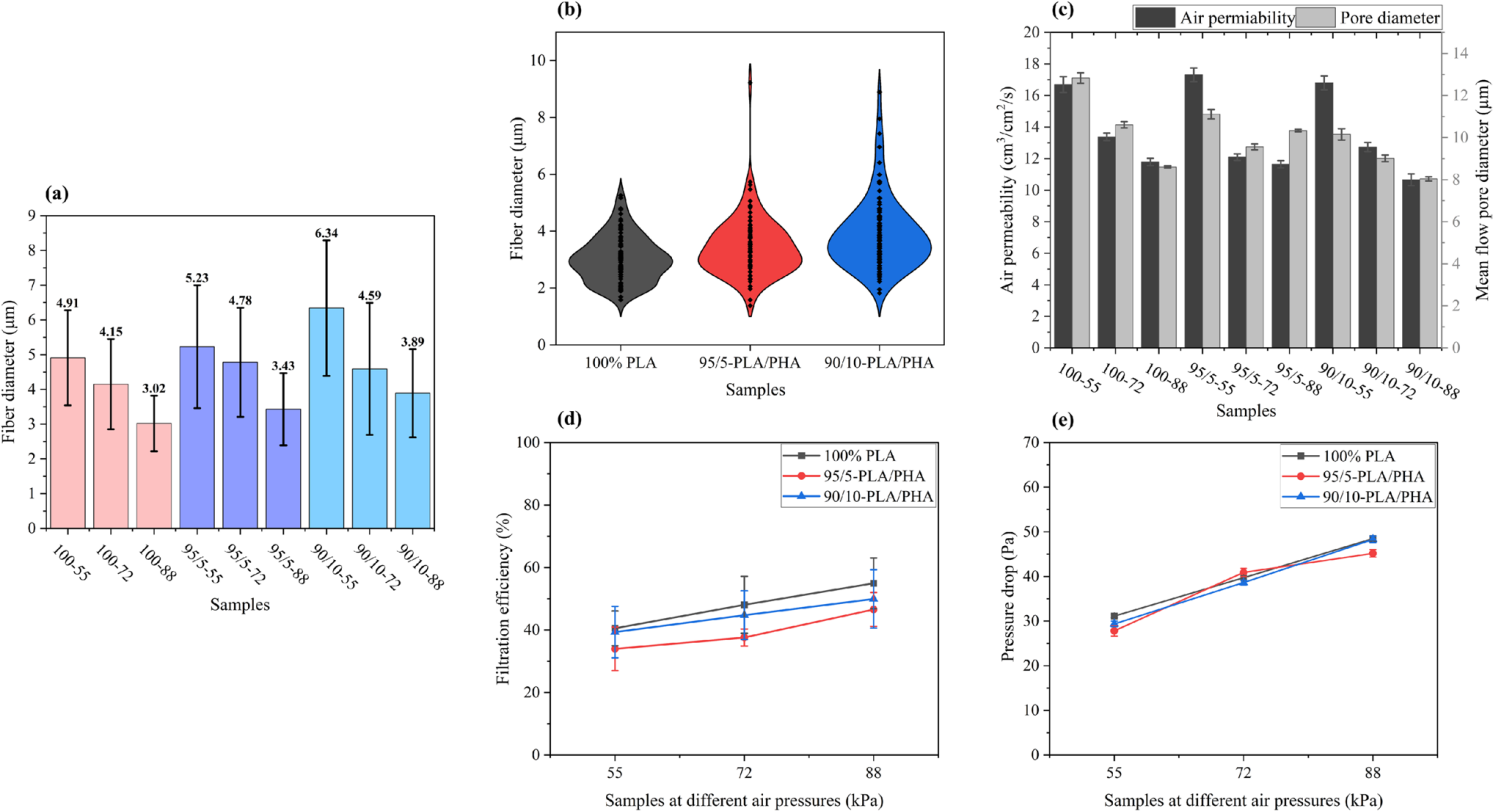
**a** Mean fiber diameter of PLA/PHA blended webs at different air pressures and polymer compositions **b** Effect of polymer composition on fiber diameters (combined violin plot, sample developed at 88 kPa air pressure) **c** Air permeability and mean flow pore diameter of developed webs at different air pressures. Effect of air pressure on **d** Filtration efficiency **e** Pressure drop of PLA and PLA/PHA blended webs.

Figure 5a demonstrates the mean fiber diameters (μm) of PLA and PLA/PHA blended melt-blown nonwovens at different air pressures (55, 72, and 88 kPa). For 100% PLA, increasing air pressure significantly decreased fiber diameter (*p* value =  < 2 × 10^–16^ ***). Multiple comparison through the Tukey HSD test demonstrated that all the comparisons were statistically significant (*p* value =  < 0.001) and the order of fiber diameters was 100–55 > 100–72 > 100–88. However, the box plot of the three different samples demonstrated the presence of more outliers in the 100–55 and 100–72 samples, and the 100–88 sample represented more uniform webs with finer diameters (Fig. S2).

Furthermore, for the 95/5-PLA/PHA samples as well, there was a significant difference (*p* value = 5.7 × 10^–16^ ***) of fiber diameters among the three different samples developed at those air pressures. However, Tukey HSD post Hoc results confirmed no significant difference in fiber diameters (*p* value = 0.087) between 95/5–55 and 95/5–72 blended webs, although 95/5–72 fibers were slightly thinner, which could be due to random variation. In contrast, 95/5–88 fibers were significantly thinner than both 95/5–52 and 95/5–72 (*p* value =  < 0.001) (Fig. S3).

In addition, for 90/10-PLA/PHA samples, highly significant differences existed between at least two of the three samples (*p* value =  < 0.001). The between-group variation (Mean Sq = 159.87) was much larger than the within-group variation (Mean Sq = 3.01). The Tukey HSD post Hoc test confirmed significant pairwise differences. 90/10–55 produced the thickest fibers, significantly thicker than both 90/10–72 (*p* value =  < 0.000001) and 90/10–88 (*p* value =  < 0.000001). 90/10–88 produced the thinnest fibers, even thinner than 90/10–72, though the difference was smaller (*p* value = 0.0130295) (Fig. S4). From all this data and analysis, higher air pressures result in more uniform webs with finer fibers.

### Effect of polymer composition on fiber diameter

Polymer composition significantly impacted the mean fiber diameters and distribution of fiber diameters. Figure 5b demonstrates the violin plot of the fiber diameter distribution of three distinct groups of samples (100% PLA, 95/5-PLA/PHA, and 90/10-PLA/PHA). The 100% PLA had a narrower distribution, which indicated less variation in fiber diameters. On the other hand, 95/5-PLA/PHA had a slightly wider spread than 100% PLA, which indicated a higher variability. 90/10-PLA/PHA had the widest distribution with more outliers, suggesting significant variability in fiber diameters. The Shapiro–Wilk test was performed to investigate whether the data follow a normal distribution of fiber diameters. All three groups failed the normality test (*p* values < 0.05). This finding was supported by the findings in the fiber diameter distribution of three separate groups of samples (Fig. S1), where there are more extreme values on one side (skewed distribution).

Therefore, one-way ANOVA was not appropriate because ANOVA assumes normality. In this scenario, the Kruskal–Wallis test (nonparametric alternative) was performed to investigate whether the distribution of fiber diameters was the same across all groups (null hypothesis, H_o_). The Kruskal–Wallis rank sum test demonstrated a significant difference in diameter distribution among the three groups at the 0.05 level of significance (Kruskal–Wallis chi-squared = 30.509, df = 2, *p* value =  < 0.00001). The Dunn’s test with Bonferroni correction was used to determine pairwise comparisons among the three groups (100% PLA, 95/5-PLA/PHA, and 90/10-PLA/PHA). The negative z-score (− 5.52) indicated that the median fiber diameter of 100% PLA was significantly lower than that of 90/10-PLA/PHA. Since the adjusted *p* value was extremely small (*p* = 1.007134 × 10^–07^), this difference was highly significant. In the case of 100% PLA and 95/5-PLA/PHA, a negative z-score (-2.88) suggested that the median fiber diameter of 100% PLA was lower than that of 95/5-PLA/PHA.

Furthermore, the adjusted *p* value was lower than 0.05, indicating a statistically significant difference, but it was less extreme than the first pairwise comparison (100% PLA and 90/10-PLA/PHA). Similarly, 90/10-PLA/PHA fibers were significantly coarser than 95/5-PLA/PHA fibers (z-score = 2.64, *p* = 0.02).

This suggested that adding PHA to PLA significantly affected the fiber diameters, with higher PHA composition leading to coarser fibers, which could be due to changes in melt rheology. Adding amorphous PHA increased the melt viscosity of the blend due to the lower MFR of the PHA. Consequently, the polymer jet was less resistant to stretching and thinning, leading to the formation of coarser fiber [28, 40]. This conclusion was further supported by the fact that when the processing temperature was increased for a 90/10 blend of PLA/PHA, finer fibers could be produced because of a reduction in apparent melt viscosity.

### Air permeability of blended webs

Air permeability is a crucial property of melt-blown nonwoven webs that significantly influences their performance in various applications, particularly in filtration, where a balance between air permeability and filtration efficiency is essential. Figure 5c demonstrates the air permeability of PLA/PHA blended melt-blown nonwoven webs at three different air pressures. As the process air pressure increased (from 55 to 88 kPa), air permeability decreased across all the samples. This suggested that higher air pressure produced finer fibers and denser webs, and reduced air permeability. Higher air pressure increased hot air stream velocity, which accelerated the molten polymer filaments exiting the die. This resulted in greater fiber stretching and attenuation, which led to a smaller fiber diameters. Smaller fiber diameters created a denser web structure with reduced pore size, which restricted the airflow and decreased air permeability. Furthermore, finer fibers occupied more volume per unit area and restricted airflow through the web [41, 42].

### Pore size distribution

Figure 5c depicts the mean flow pore diameter of 100% PLA and the PLA/PHA blended webs processed at different air pressures. Higher air pressure intensified polymer attenuation, producing finer fibers with narrower diameter distributions. These finer fibers formed denser and more compact networks, and reduced pore dimensions [42, 43].

For 100% PLA, the largest pore (12.8 μm**)** structure was obtained at 55 kPa air pressure due to inconsistent fiber entanglement. However, the smallest pores (8.6 μm) were obtained at 88 kPa air pressure due to higher air turbulence that compresses fibers, resulting in reduced pore sizes and improved uniformity. Incorporation of 5% PHA behaved similarly to pure PLA. However, samples with 10% PHA demonstrated smaller and more uniform pores. These observed results were due to the combination of both fiber diameter and packing of fibers, which changed due to the softness of the fibers.

### Effect of air pressure on barrier properties

Air pressure significantly influences fiber morphology and web structure in melt-blown processes, directly affecting filtration efficiency and pressure drop. Higher air pressure during processing enhances the filtration efficiency of the developed webs by reducing fiber diameters and consequently creates denser webs [41].

For 100% PLA, there was a significant difference in mean filtration efficiency (*p* value = 0.00943** at 0.05 significance level) among the sample processed in different air pressures. Filtration efficiency significantly increased with increasing air pressure from 55 to 72 kPa (*p* value = 0.0348677) and from 55 to 88 kPa (*p* value = 0.0124952). However, filtration efficiency didn’t significantly change at 72 and 88 kPa (*p* value = 0.8982169). Furthermore, there was a substantial difference in pressure drop (*p* value =  < 0.00001) among the samples. Because of a denser web structure, the pressure drop of the developed webs increased with increasing air pressure from 55 to 72 kPa (*p* value =  < 0.000001), 55 kPa to 88 kPa (*p* value =  < 0.000001), and even from 72 to 88 kPa (*p* value =  < 0.000001). Higher air pressure during melt blowing produced finer fibers, which increased web density and reduced pore size. This led to higher resistance to air flow, which was reflected in greater pressure drop.

In addition, for 95/5-PLA/PHA, there was a significant difference in filtration efficiency (*p* value = 0.000771 ***) of the samples developed at different air pressures. Higher air pressure (88 kPa) led to significantly higher filtration efficiency compared to both 55 kPa (*p* value = 0.0005588) and 72 kPa (*p* value = 0.0318413). However, increasing air pressure from 55 to 72 kPa did not reliably improve filtration efficiency (*p* value = 0.2543009). In contrast, raising air pressure significantly increased pressure drop by 12.7 units (55–72 kPa), 17.2 units (55 to 88 kPa), and 4.5 units (72–88 kPa), all significant differences (*p* < 0.000001).

For 90/10-PLA/PHA, there were no statistically significant differences in filtration efficiency among the samples developed at three air pressure groups (*p* value = 0.0653). However, the increase in pressure drop was significant (*p* value =  < 0.00001). Most importantly, for all the samples, pressure drop remained < 50 Pa. Figure 5d and e depicts the effect of air pressure on the barrier properties of the blended webs.

### Correlation between fiber diameters and barrier properties

**Figure 6:**
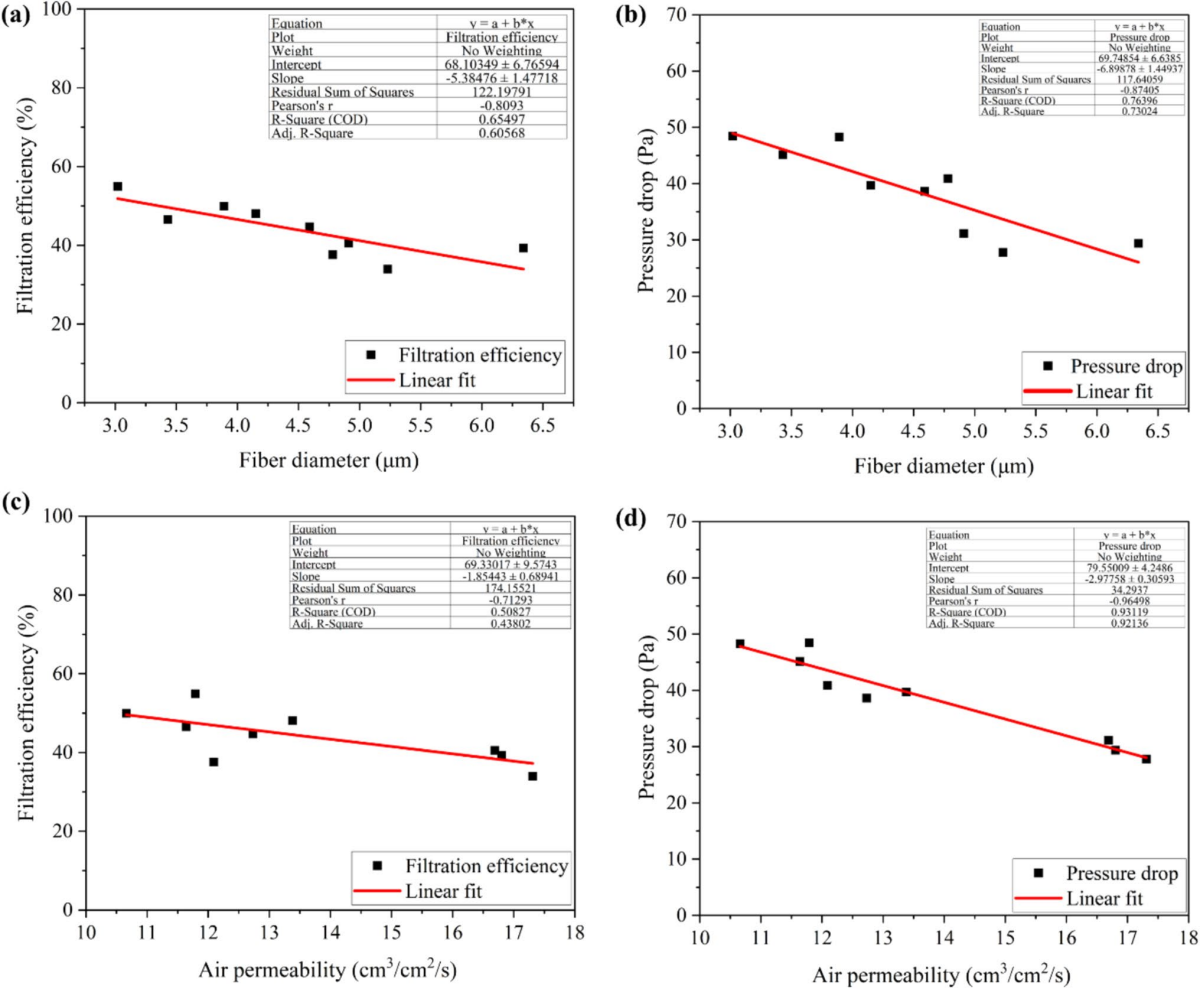
Correlation of fiber diameters with **a** Filtration efficiency and **b** Pressure drop. Correlation between **c** Air permeability and filtration efficiency and **d** Air permeability and pressure drop.

The fiber diameter vs. filtration efficiency plot demonstrated a strong negative correlation, as indicated by the correlation coefficient *r* = − 0.81. On the other hand, for the pressure drop, it was -0.87 (Fig. 6a and b). Each point represents a sample developed in this experiment. This indicated that as the fiber diameter increased, mechanical filtration efficiency and pressure drop decreased. This was because finer fibers increased the specific surface area for a given mass of material. Furthermore, nonwoven webs with finer fiber diameters efficiently captured particles, leading to enhanced adsorption and higher filtration efficiency [41, 44]. Furthermore, particle collection efficiency by diffusion and interception increased as the fiber diameters decreased. Kim et al. [45] developed three nanofiber filters with diameters of 100, 201, and 306 nm with the sample physical properties (packing density, thickness, and basis weight) and observed that smaller fibers led to higher particle capture and pressure drop. In addition, nonwoven webs with finer fibers formed a denser and more intricate network with smaller pores. This structure physically blocked more particles from passing through (distance between fibers is reduced and the path for particles is more tortuous) and improved mechanical filtration efficiency and pressure drop [41, 45].

### Correlation between air permeability and barrier properties

Figure 6c demonstrates the correlation between air permeability and filtration efficiency. There was a strong negative correlation (− 0.71) between air permeability and filtration efficiency of melt-blown webs. As air permeability increased, filtration efficiency decreased. This was because higher air permeability indicated a more open web structure, allowing more air—and consequently more aerosol particles—to pass through, which reduced the material's ability to capture aerosol particles [6, 41].

Furthermore, there was a strong inverse correlation (− 0.96) between air permeability and pressure drop (Fig. 6d). This relationship arises because air permeability reflects how easily air flows through the materials, while pressure drop measures the resistance to air flow. Nonwoven webs with higher air permeability demonstrated lower pressure drop and vice versa. Higher air permeability was due to a larger pore size in the webs and reduced pressure drop. In addition, higher air pressure during processing attenuates fibers more, reduces their diameter and pore size, which lowers air permeability and raises pressure drop [46].

### Surface potential of the corona-charged webs

For further analysis of corona charging impact, 100–88, 95/5–88, and 90/10–88 were charged at –50 kV using a dual-bar corona charging system. Those three samples were selected because of their finer fiber diameters from corresponding groups and the best mechanical filtration efficiency.

As produced samples indicated random positive and negative surface potential through friction/triboelectric effects between fibers and manufacturing equipment. However, after charging, negative corona discharges flooded the webs with high-density electrons and negative ions, and only negative potential was observed throughout the surface. While charging, the high-voltage power supply (− 50 kV) was connected to the needle electrode, which was placed above the grounded collector cylinder. Nonwoven webs were passed between them. High applied voltage created a strong electric field around the electrode tip and ionized the air molecules near the electron tips and released electrons. These electrons collided with neutral air molecules and formed negative ions. Due to the strong potential differences, negative ions migrated toward the grounded electrode. The webs intercepted the ions and became electrostatically charged [10, 47, 48].

**Figure 7:**
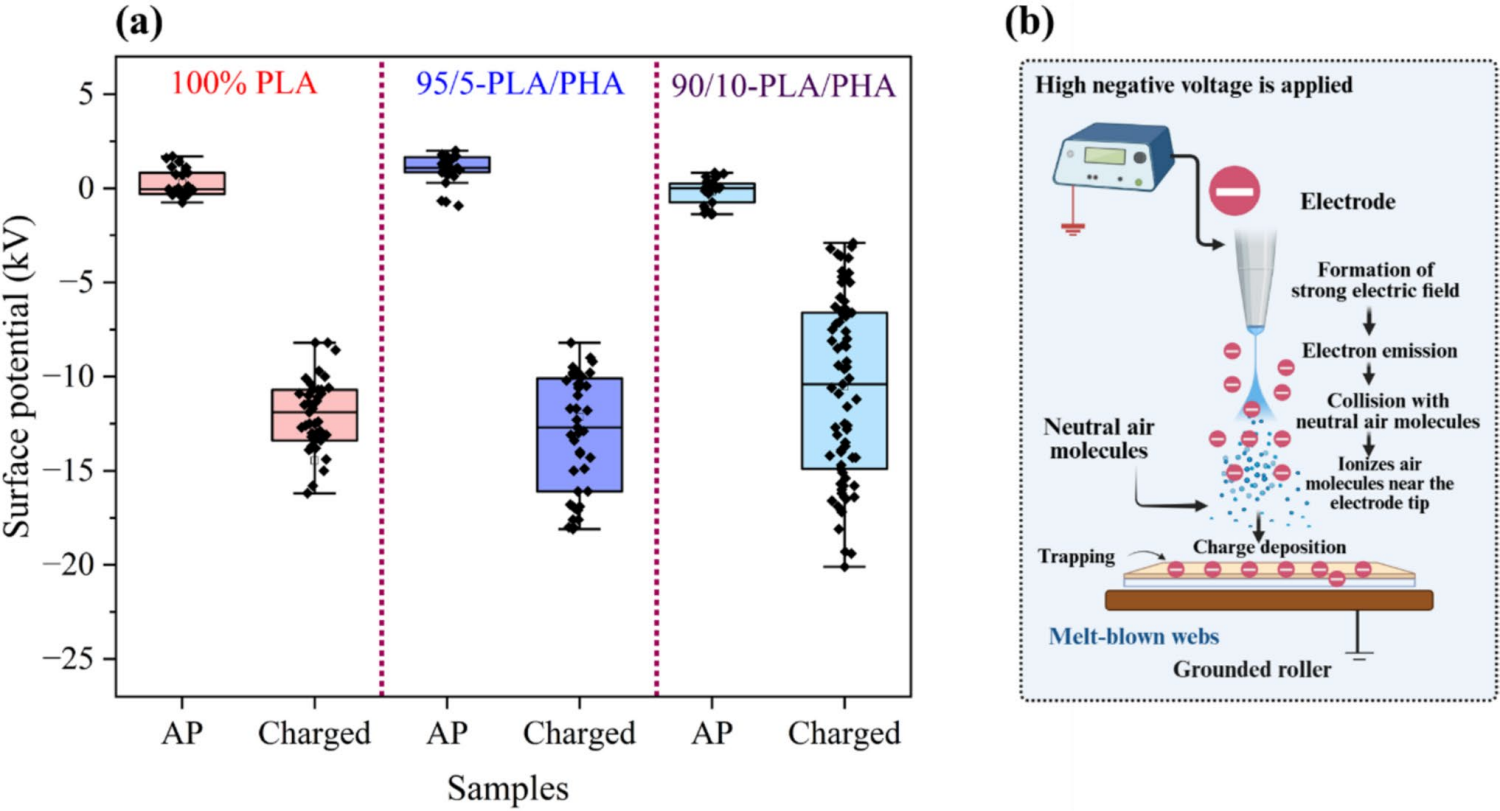
**a** Comparison of surface potential of corona-charged pure PLA and PLA/PHA blended webs. **b** Charge generation mechanism during negative corona charging.

There is no statistically significant difference in surface potential of the face and back side of pure PLA (*p* value = 0.618), 95/5-PLA/PHA (*p* value = 0.697), and 90/10-PLA/PHA (*p* value = 0.8318) after corona charging. However, there was a significant difference in the surface potential among the three groups of samples (*p* value = 0.002). 100% PLA demonstrated the highest mean surface potential (–14.47 kV) among the three groups (–12.15 kV for 95/5-PLA/PHA and –10.54 kV for 90/10-PLA/PHA). The increase was due to the smallest fiber diameter of the 100% PLA web among the samples. As fiber diameter decreased, surface area also increased, along with surface potential (Fig. 7a). This finding was supported by literature [49].

### Effect of corona charging on barrier properties

Before corona charging, the nonwoven webs rely solely on mechanical filtration (inertial impaction, direct interception, Brownian diffusion, and gravitational settling), and mechanical filtration efficiency is highly sensitive to small variations in fiber diameter, web uniformity, pore size, and local defects at different places of the webs. Therefore, developed webs had a higher standard deviation in filtration efficiency before corona charging [50].

**Figure 8:**
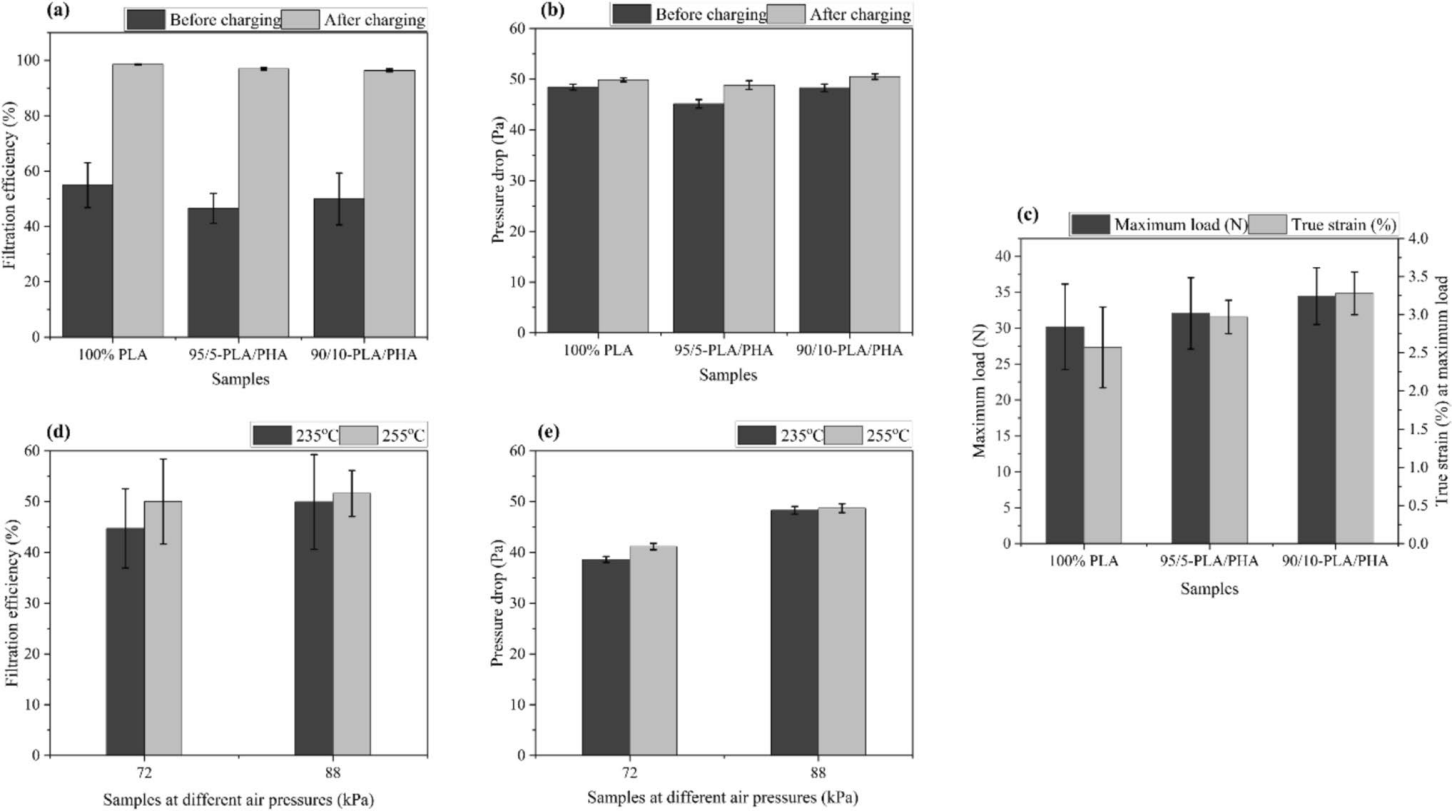
Effect of negative corona charging on **a** Filtration efficiency and **b** pressure drop of PLA/PHA blended nonwoven webs **c** Mechanical performance (tensile properties) of PLA/PHA blended webs. Sample developed at 88 kPa air pressure. Effect of melt temperatures (235 and 255 °C) on **d** Filtration efficiency and **e** Pressure drop of developed webs.

However, after charging, the introduction of electrostatic charge provided a much more consistent and reliable capture mechanism, dramatically reducing variability. Furthermore, for all three samples, filtration efficiency increased significantly (*p* value = 2.374 × 10^–07^, 4.399 × 10^–09^, 3.77 × 10^–07^, respectively) after charging, and filtration efficiency after charging was > 96% for all sample types. During corona charging, negative ions were deposited on the nonwoven surface and became trapped within the materials as trapped charges and imparted an electrostatic capture mechanism, which consequently increased the filtration efficiency of the webs (Fig. 7b) [51–53]. However, among the three samples, 100% PLA demonstrated the highest filtration efficiency, and there is a highly significant difference among the samples (*p* value = 1.81 × 10^–06^ ***). The increase was due to the smallest fiber diameter of 100% PLA webs among the samples. PLA/PHA blend showed a decreasing trend of filtration efficiency since the incorporation of PHA increased the fiber diameter. As fiber diameter decreased, surface area and mechanical filtration efficiency increased, along with surface potential and electrostatic filtration efficiency. This finding was supported by other studies in literature [49]. Figure 8a depicts the effect of corona charging on the filtration efficiency of pure PLA and PLA/PHA blended webs.

However, after corona charging, pressure drop (Fig. 8b) also significantly improved (*p* value = 0.000309, 4.822 × 10^–07^, 0.0002848, respectively for 100% PLA, 95/5-PLA/PHA, and 90/10-PLA/PHA). However, the overall pressure drop was below 51 Pa. The increase in pressure drop after corona charging might be due to the change in fiber surface properties or packing density while charging. Calisir et al. [48] explored a 4.53% increase in pressure drop after corona charging of PVDF nano/microfibrous filter mats [48]. After corona charging, QF increased for all samples. Among different samples, 100% PLA demonstrated the highest QF, suggesting it had the most favorable combination of filtration efficiency and pressure drop. A higher quality factor indicates better overall filtration performance, balancing high filtration efficiency with low-pressure drop. Table 4 summarizes the QF of the developed webs.

**Table 4:** QF of the developed webs (pure PLA and PLA/PHA blended webs)

Samples	QF (Pa^−1^)	
	Before charging	After charging
100% PLA	0.0165	0.0849
95/5-PLA/PHA	0.0139	0.0716
90/10-PLA/PHA	0.0144	0.0659

### Tensile properties

Figure 8c demonstrates the tensile properties of pure PLA and PLA/PHA blended webs. The incorporation of PHA into PLA webs enhanced the tensile properties, particularly true strain (%) and maximum load (N) to break the specimen in the machine direction. Pure PLA is inherently brittle (low true strain = 2.5%) due to high glass transition temperature (Tg ~ 58 °C), low modulus of elasticity, and rigid polymer chains with limited mobility. The flexible chain of PHA increased free volume, enabling polymer chain slippage. Furthermore, amorphous PHA under stress deflects cracks, dissipating energy and delaying fractures to improve the impact strength of the blended webs. Therefore, incorporation of PHA significantly improved the maximum load to break the specimen (*p* value—8.34 × 10^–06^ ***). The 90/10-PLA/PHA blend demonstrated ~ 14.1% improvement in the maximum force to break the specimen (toughening). Furthermore, true strain (%) at maximum load of the composite webs also increased significantly (~ 27.6%) due to plasticization effect (*p* value = 6.92 × 10^–06^ ***) [28].

Bartczak et al. [54] also demonstrated a significant increase in the ultimate elongation (above 20%) for PLA/aPHB blend (8:2) [54]. Noda et al. [55] investigated that adding 10% (wt.%) PHA copolymer greatly increased the blend’s toughness-making it 10 times higher than that of pure PLA. The enhanced toughness was attributed to the fine dispersion of PHA in the PLA matrix and reduced crystallinity due to the amorphous state of the PHA [55].

### Effect of air pressure on tensile properties

Figure S5 depicts the effect of processing conditions on the mechanical performance of the developed webs. Increasing air pressure during processing had a significant impact on mechanical performance. Increasing air pressure from 55 to 88 kPa led to higher mean maximum force for most sample compositions. For pure PLA, the mean maximum force was the lowest at 72 kPa (28.3 N) and highest at 88 kPa (30.2 N). The increase was modest, but higher air pressure improved tensile strength. For 95/5-PLA/PHA, tensile strength was consistent across the air pressures (32.1 to 32.9 N), with a slight peak at 72 kPa. For 90/10-PLA/PHA, tensile strength increased more clearly with pressure. As air pressure increased, the molten polymer was attenuated more strongly, resulting in fibers with smaller fiber diameters. Finer fibers increased the surface area, number of fiber–fiber contact points (effective fiber bonding), and improved web uniformity, which increased mechanical strength [41, 56]. While strength increased, the true strain at break (ductility) decreased with higher air pressure. This was because the webs became denser and stiffer and less able to stretch before breaking, reducing ductility [57].

### Effect of melt temperature

**Figure 9:**
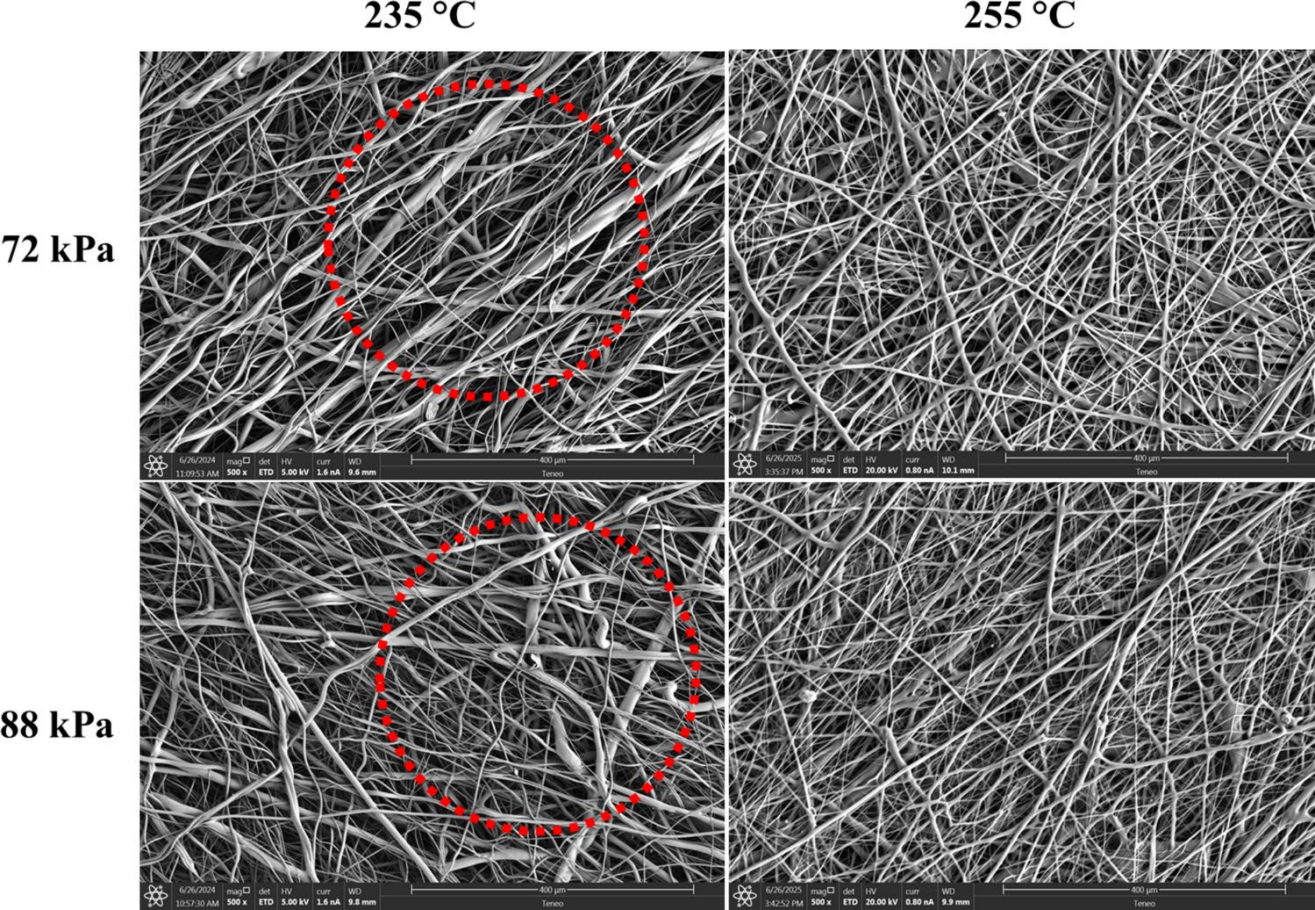
SEM images and distribution of fiber diameters at different melt temperatures (magnification—500 × , ETD detector).

Among different nonwoven webs, 90/10–88 webs demonstrated the lowest filtration efficiency after corona charging (96.4%). Therefore, the impact of melt temperature was investigated for this sample. The resulting web characteristics were significantly impacted by the melt temperature (Fig. 8d and e). Table 5 summarizes the effect of melting temperature on the performance of PLA/PHA blended webs. Higher melt temperatures lowered the viscosity of the polymers, making it easier to stretch and elongate the fibers during formation. As a result, the fibers produced were finer in diameter. Denser webs with greater surface area demonstrated improved mechanical filtration efficiency and pressure drop (Fig. 9) [58–60]. Dzierzkowska et al. [61] obtained the finest fibers (PLA melt-blown nonwovens scaffold) at the die temperature above 260 °C [59, 61]. According to Wang et al. [62], the diameter of nonwovens produced from PLA/PEG/SDS microfibers was mostly dispersed between 4 and 10 μm at a die temperature of 210 °C. When the PLA melted to 225 °C, the resulting fiber diameter dropped to less than 4 μm, increasing the density and quantity of inter-fiber apertures [62, 63]. Yesil et al. [64] observed average fiber diameter of PP melt-blown nonwovens slightly decreased when the die temperature increased from 240 to 255 °C [64]. This finding was supported by many other researchers as well [59, 65, 66].

**Table 5:** Effect of melt temperature (processing 10 wt.% PHA blend, processed at higher melt temperature) (at 32 L/min)

Melt temperatures	Filtration efficiency (%)		Pressure drop (Pa)	
	Before charging	After charging	Before charging	After charging
255 °C	51.6 ± 4.54	99.0 ± 0.23	48.7 ± 0.09	51.9 ± 0.06
235 °C	49.9 ± 9.34	96.4 ± 0.52	48.3 ± 0.08	50.5 ± 0.05

To investigate the effect of corona charging on barrier properties of the developed webs at higher melt temperature, sample 90/10–88 was corona charged negatively (applied voltage: − 50 kV, electrode-sample distance: 7 cm, and speed: 2 m/min). Developed webs demonstrated a significant increase in filtration efficiency (*p* value =  < 0.0001) after corona charging and achieved > 99% filtration efficiency with a marginal increase in pressure drop at 32 L/min (Table 5).

## Conclusions

This study demonstrates the successful development of bio-based, biodegradable melt-blown nonwovens from PLA and PLA/PHA blends for high-efficiency air filtration. The fabricated webs exhibited uniform structure with fiber diameters of 3–7 µm, while PHA incorporation increased fiber diameter due to higher melt viscosity without inducing chemical interactions between components. Thermal analysis indicated reduced *T*_g_, *T*_c_, and slightly lower melting temperatures, confirming the plasticizing effect of PHA and its influence on PLA crystallinity and processability. Furthermore, PLA/PHA blended webs showed improved mechanical performance, with ~ 14.1% higher strength and ~ 27.6% greater strain at maximum load, reflecting enhanced toughness due to increased free volume and polymer chain slippage with amorphous PHA incorporation. In addition, the developed webs achieved > 96% PM_0.3_ filtration efficiency with low-pressure drop (< 51 Pa) after corona charging, and further optimization of melt temperature enabled efficiencies exceeding 99% by improving fiber uniformity and web structure. Overall, these findings highlight PLA/PHA blended melt-blown nonwovens as a promising sustainable alternative to conventional PP-based filtration media, combining high filtration performance with improved mechanical properties and biodegradability.

## Supplementary Information

Below is the link to the electronic supplementary material.

Supplementary file1 (DOCX 443 KB)

## Data Availability

The data supporting the findings of this study are available from the corresponding authors upon reasonable request.

## Abbreviations

PM: Particulate matter
PP: Polypropylene
PE: Polyethylene
PET: Polyethylene terephthalate
EBS: N, N'-ethylene bis-stearamide
PLA: Polylactic acid
PHA: Polyhydroxyalkanoate
PHB: Polyhydroxybutyrate
PHBV: Poly(3-hydroxybutyrate-co-3-hydroxyvalerate)
PBS: Polybutylene succinate
DCD: Die-to-collector distance
MFR: Melt flow rate
QF: Quality factor
PVDF: Polyvinylidene fluoride
PEG: Polyethylene glycol
SDS: Sodium dodecyl sulfate
