# Supplementary material for: Processing, structure, and properties of PLA/PHA blended melt-blown nonwoven webs for filter media applications
Source: J Mater Sci. 2026 Jun 24;61(31):22797–818. doi: 10.1007/s10853-026-13145-5 (PMC13328327; doi:10.1007/s10853-026-13145-5)
Supplement: Supplementary file 1 — Supplementary file1 (DOCX 443 KB) [file 10853_2026_13145_MOESM1_ESM.docx]

**Processing, Structure, and Properties of PLA/PHA Blended Melt-blown Nonwoven Webs for Filter Media Applications**

Avik Kumar Dhar^1^, Ivan Moldavchuk^1^, Maitry Bhattacharjee^2^, Joe Nageotte^1^, Gajanan Bhat^1^, & Sudhagar Mani^2^

^1^Department of Textiles, Merchandising, and Interiors, University of Georgia, 321 Dawson Hall, 305 Sanford Drive, Athens GA 30602, USA

^2^School of Chemical, Materials, and Biomedical Engineering, University of Georgia, Athens, GA, 30605, USA

**Table S1** Processing conditions of PLA and PLA/PHA blended webs

| Parameters | Value |
| --- | --- |
| Melt/die temperature | 235°C |
| Air temperature | 260°C |
| Die-to-collector distance | 14 cm |
| Collector drum speed (rpm) | 3 |
| Die | 10 holes per cm |
| Die hole diameter | 0.03556 cm |
| Air pressures | 55, 72, and 88 kPa |
| Polymer throughput | 0.28 g/h/m |


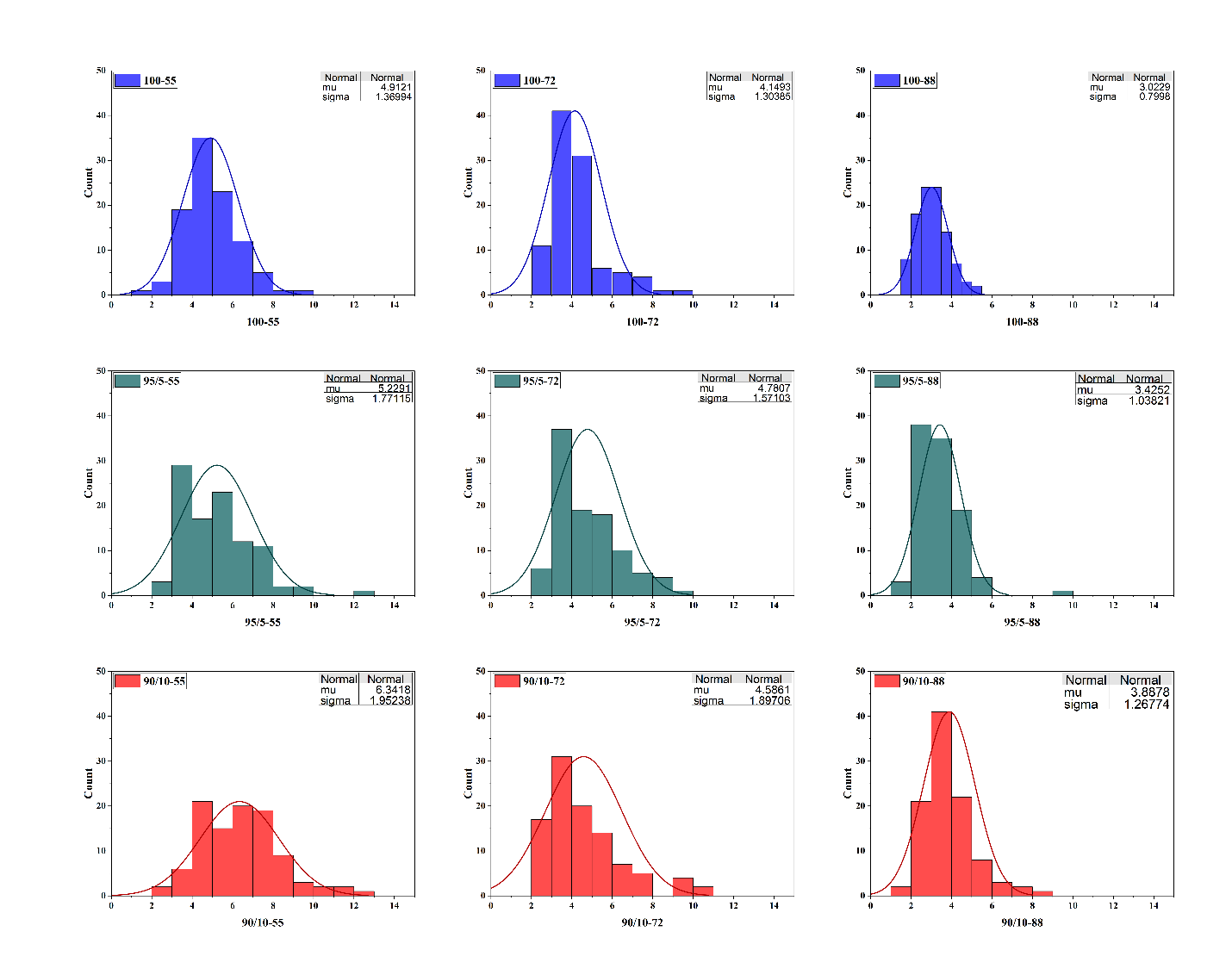


**Figure S1** Distribution of fiber diameter of PLA/PHA blended webs at different air pressures (55, 72, and 88 kPa), diameter in µm.


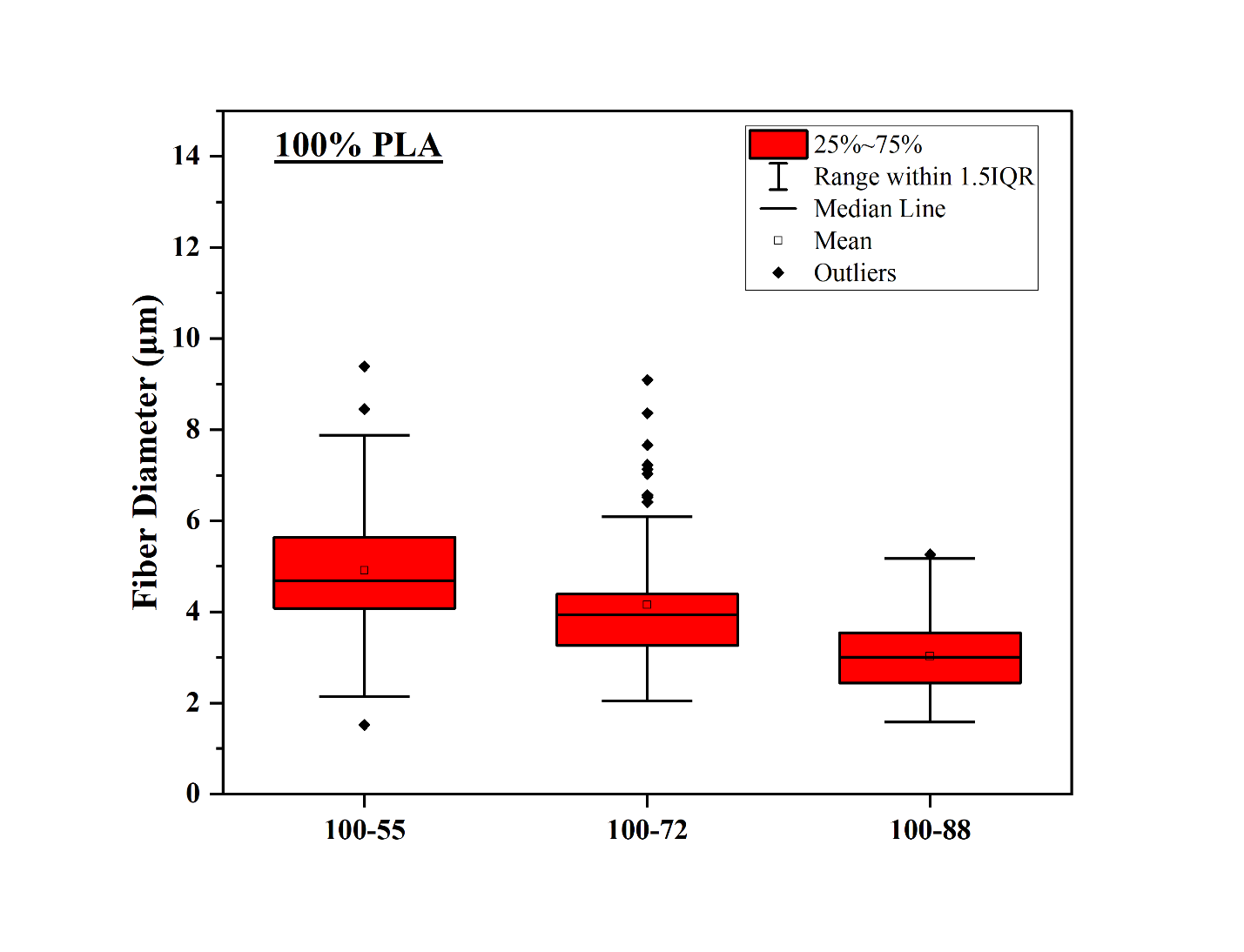
**Figure S2** Boxplot of fiber diameters (μm) of 100% PLA melt-blown webs developed at different air pressures (55, 72, and 88 kPa).


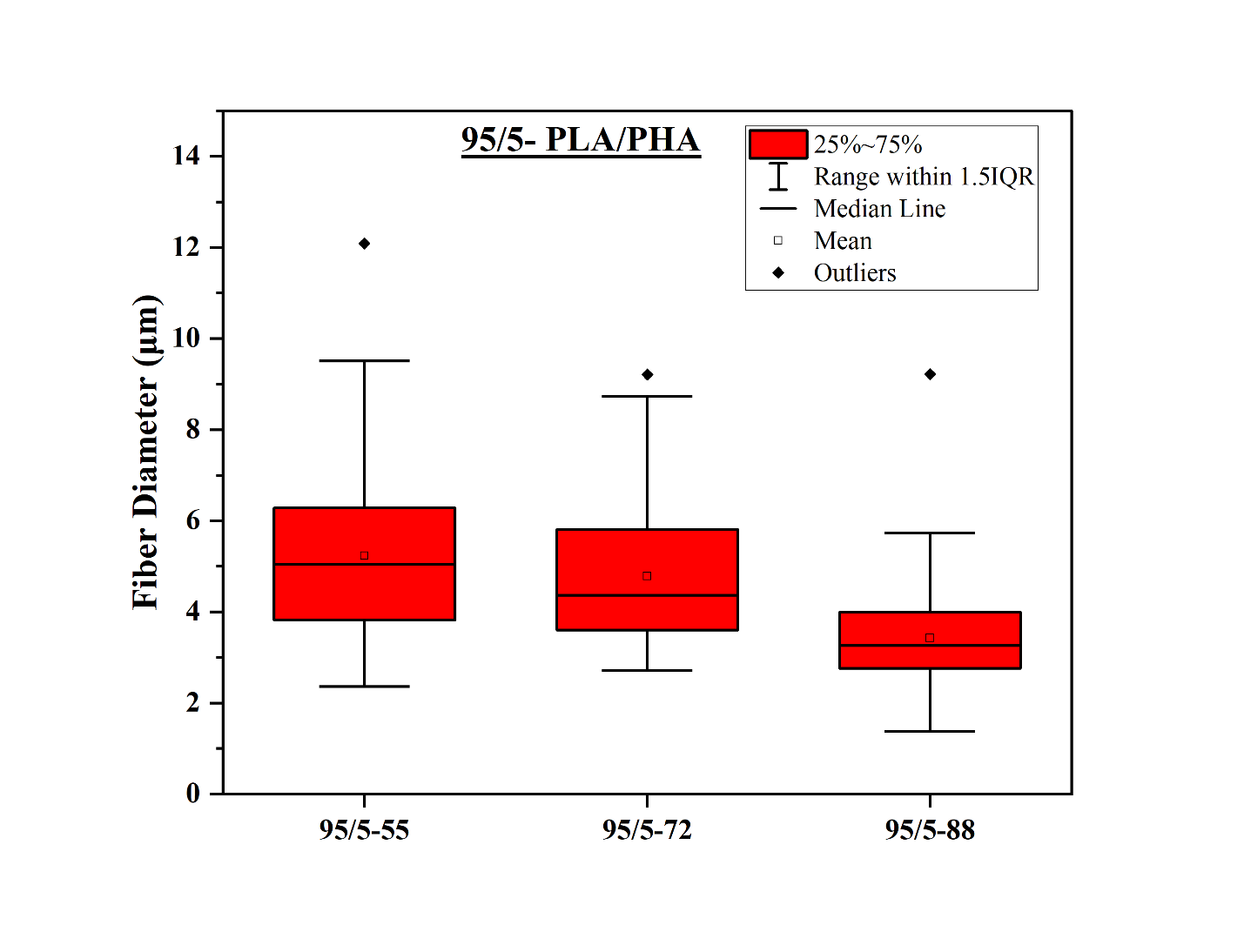
**Figure S3** Boxplot of fiber diameters (μm) of 95/5-PLA/PHA melt-blown webs developed at different air pressures (55, 72, and 88 kPa).


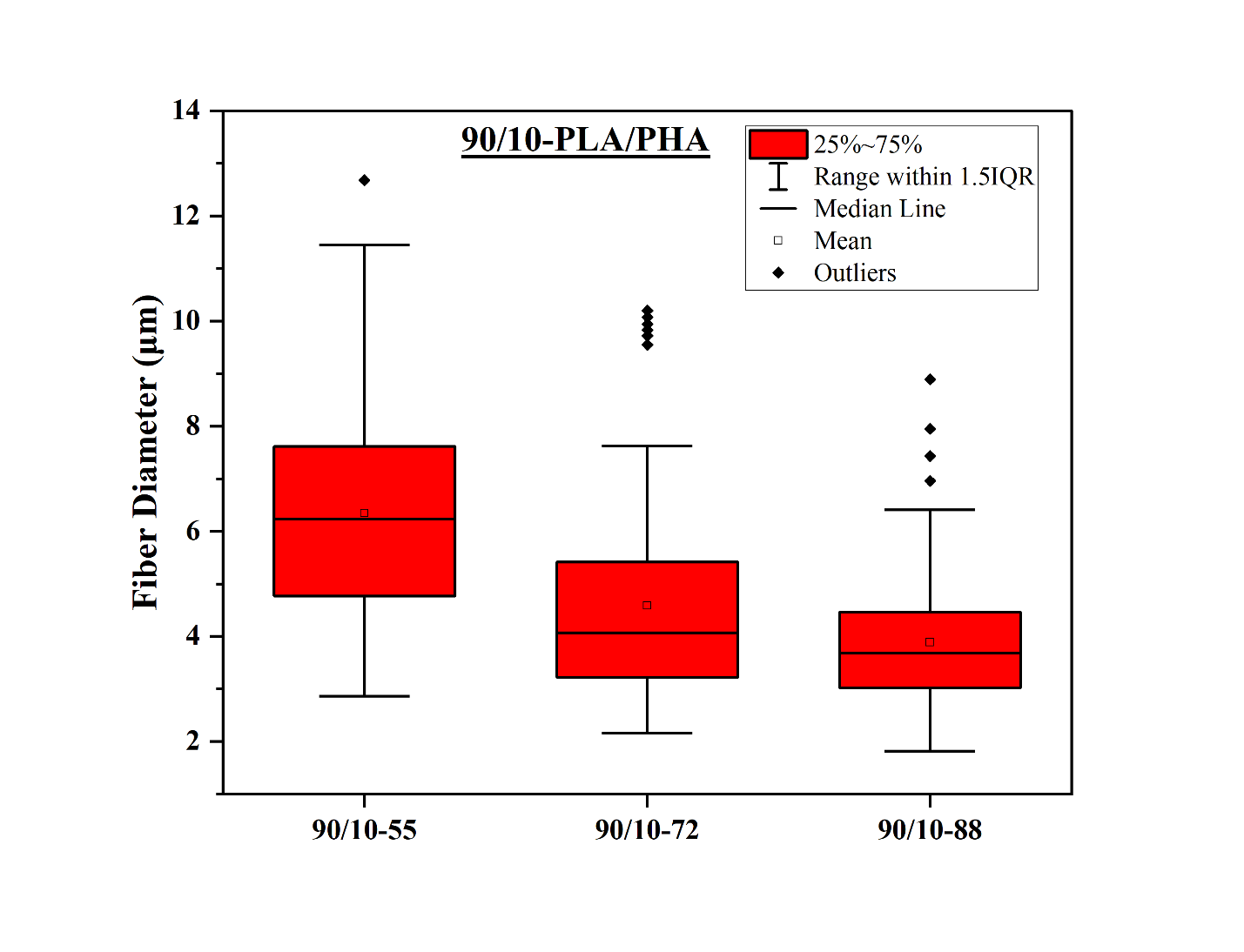
**Figure S4** Boxplot of fiber diameters (μm) of 90/10-PLA/PHA melt-blown webs developed at different air pressures (55, 72, and 88 kPa).


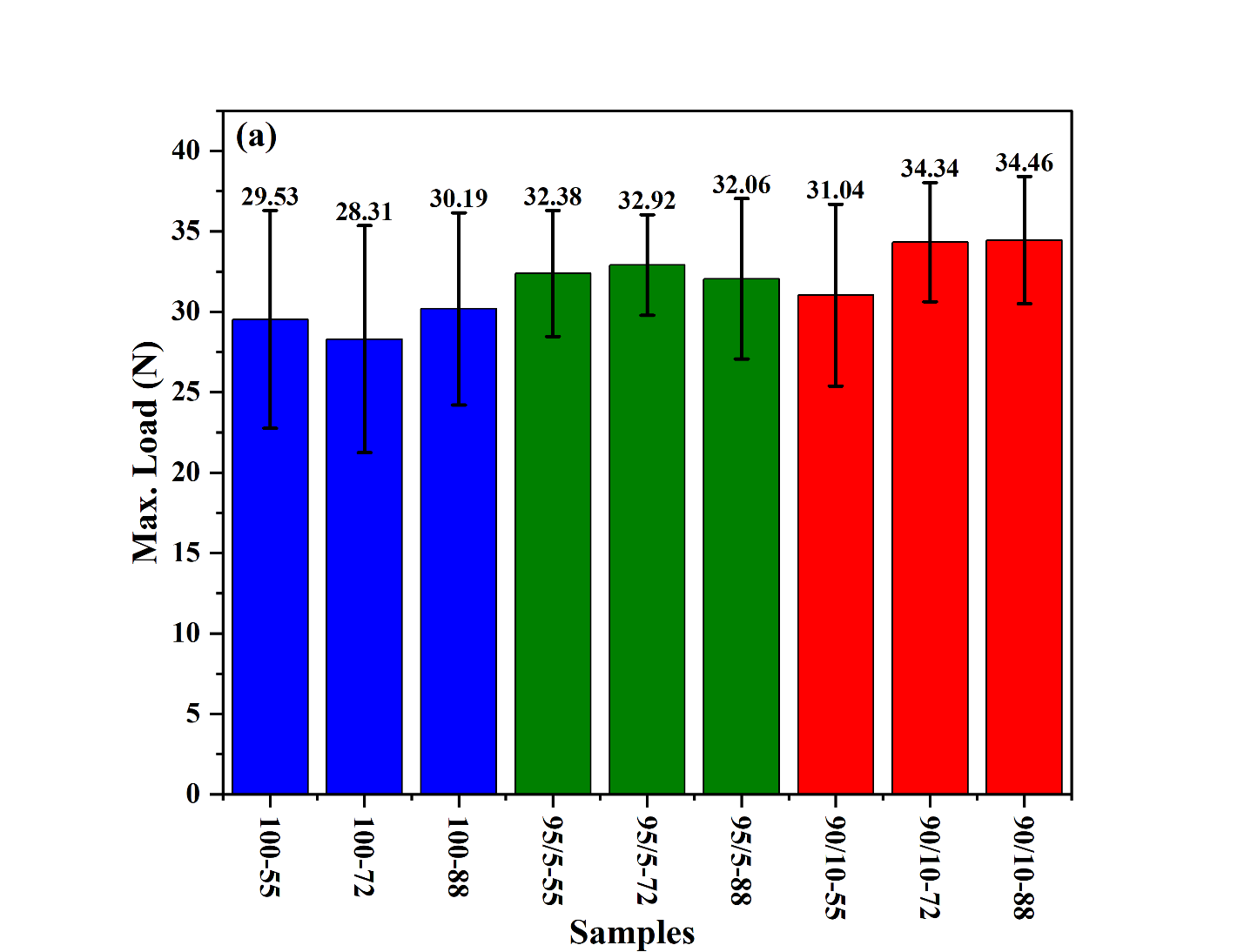


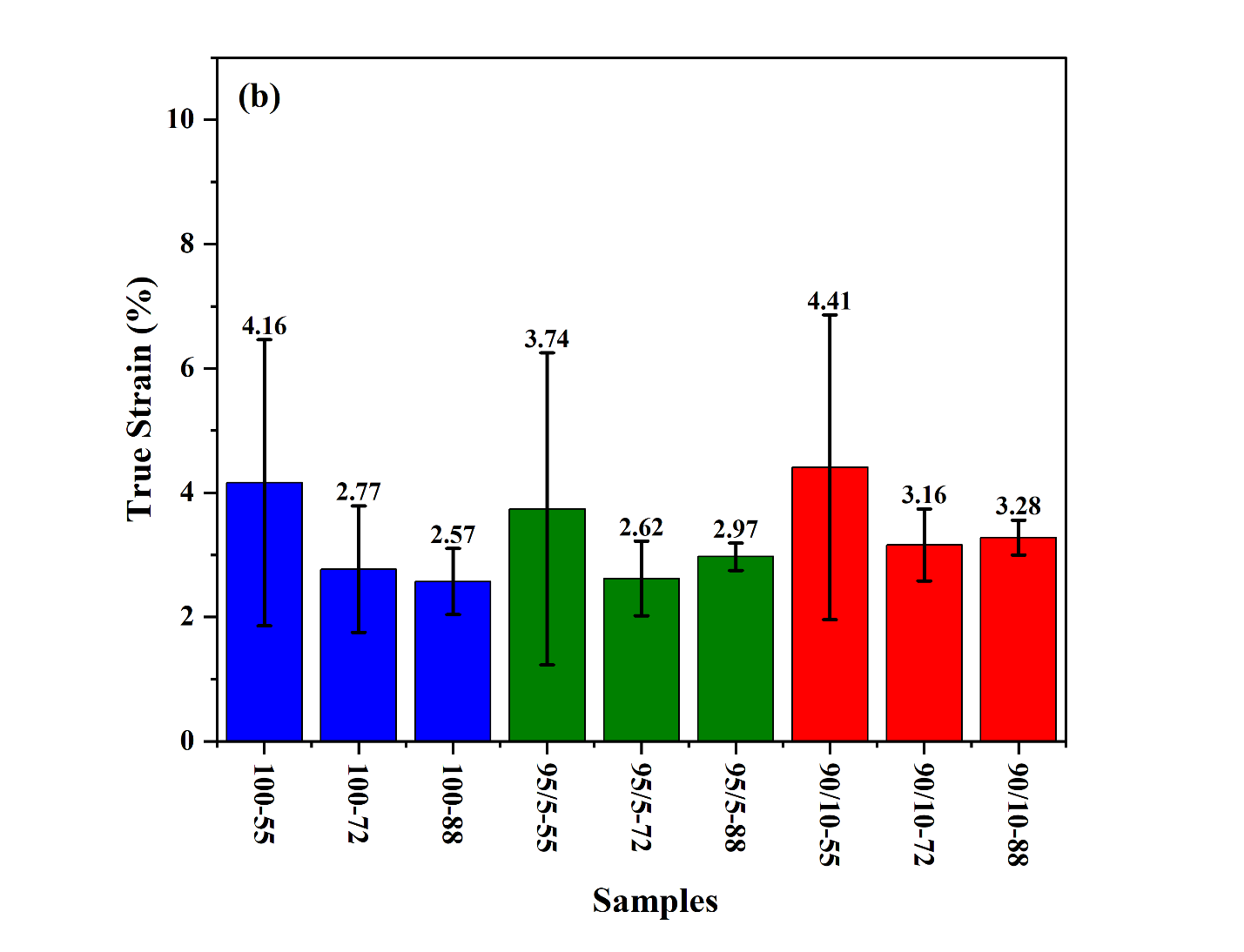


**Figure S5** Effect of air pressure on the mechanical performance of webs (a) maximum load to break (N) and (b) true strain (%).
